# Biophysical studies of cancer cells’ traverse-vessel behaviors under different pressures revealed cells’ motion state transition

**DOI:** 10.1038/s41598-022-11047-5

**Published:** 2022-05-05

**Authors:** Xiao Li, Jialin Shi, Ziqing Gao, Jian Xu, Shujing Wang, Xin Li, Qi Ouyang, Chunxiong Luo

**Affiliations:** 1 The State Key Laboratory for Artificial Microstructures and Mesoscopic Physics, School of Physics, Peking University, Beijing, China; 2 Center for Quantitative Biology, Academy for Advanced Interdisciplinary Studies, Peking University, Beijing, China; 3 Peking-Tsinghua Center for Life Sciences, Peking University, Beijing, China; 4 Wenzhou Institute University of Chinese Academy of Sciences, Wenzhou, Zhejiang China; 5 Oujiang Laboratory, Wenzhou, Zhejiang China

**Keywords:** Biological physics, Biophysics

## Abstract

Circulating tumor cells (CTCs) survive in the bloodstream and then seed and invade to foster tumor metastasis. The arrest of cancer cells is favored by permissive flow forces and geometrical constraints. Through the use of high-throughput microfluidic devices designed to mimic capillary-sized vessels, we applied pressure differences to cancer cells (MCF-7 cell line) and recorded the cell traverse-vessel behaviors. Our results showed that cancer cells transform from a Newtonian droplet state to an adhesion/migration state when cancer cells traverse artificial vessels. To explain these phenomena, a modified Newtonian droplet model was also proposed. These phenomena and the modified model may reveal how CTCs in the blood seed and invade vessels under suitable conditions.

## Introduction

Cancer metastasis is the key cause of cancer mortality^1^. Primary tumors release circulating tumor cells (CTCs) into the circulatory and lymphatic systems to colonize distant organs, which causes metastasis. To colonize distant organs, cancer cells must be able to overcome the mechanical pressures imposed by architectural constraints of tissues, squeeze through capillary-sized microvessels, and adhere/seed at special locations within their lifespan. These events in metastatic processes involve the interplay between chemical signals and physical interactions. Chemical signals have long been studied for metastasis^2,3^. However, more research on physical interactions needs to be conducted. Research on the mechanical properties of cancer cells might help in understanding the biophysical mechanisms of cancer metastasis and provide some insights for cancer therapies.

Many specialized tools have been developed to measure the mechanical properties of single cancer cells, such as atomic force microscopy (AFM), optical tweezers, magnetic twisting cytometry and micropipettes. Depending on different spatiotemporal scales and different cell stages, the mechanical properties measured using these methods might vary by one to three orders of magnitude, even for the same type of cells^4,5^. As a major approach to study the mechanical properties of single cells in both the suspension stage and the adherence stage, conventional micropipette aspiration has been implemented in many fields of cell mechanics for many years^6,7^. Nevertheless, this traditional method needs skilled laboratorians to operate one cell at a time. In recent years, microfluidic-based methods for cell mechanics have been developed quite rapidly. Compared to other existing methods, microfluidic assays are high throughput and easy to implement. Integrated with time-lapse microscopes, these assays could produce high-resolution data in an automated manner^8–14^. These microfluidic-based methods not only facilitate the studies of cell mechanics but also aid in understanding the biophysical process cancer cells use to transfer in artificial blood vessels.

There have been many experiments in vivo and in vitro to study the dynamic behaviors of cancer cells in the actual or artificial blood vessels, and the related mechanisms have been partially elucidated including the roles of integrin β1 in the extravasation and the reversible reorganization of cell cluster in the capillary-sized microfluidic channels^14–16^. However, to date, most of these studies have focused on the mechanical properties of single cancer cells or cell clusters responding to high mechanical stresses on timescales of one to ten seconds^4,8,10–14^, and research on the dynamic behaviors of cells in confinement places too much emphasis on cell migration without external mechanical signals^17–20^. In fact, human physiological systems are intrinsically complex. For the circulatory system, normal blood pressure ranges from 3 to 120 mmHg^21^. However, the physiological pressure inside the lymph capillary is approximately 1–3 mmHg^22^. During metastasis, it takes hours or days for cancer cells to invade and traverse through blood and lymph capillaries^23,24^. Over long timescales, it is likely that the CTC stages might change while traveling in vessels, which influences their behavioral patterns. From this perspective, research on the dynamic behaviors of single cells in vessels exerting low pressure might help us find the critical piece in the puzzle.

In this work, a new microfluidic device was engineered to capture single cells in a capillary-shaped vessel array, and then designed pressures were applied to these cells. In one chip, we studied the dynamic traverse-vessel behaviors of tens of single cancer cells (MCF-7 cell line) with four designed low pressures for hours. Our results demonstrate that cancer cells could transform their state from a Newtonian droplet state to an adhesion/migration state over time when they were trapped in vessels. During these transformations, the apparent viscosities of cancer cells increased as the applied pressure decreased. The critical transition of the Newtonian droplet state to the adhesion/migration state was theoretically analyzed and experimentally confirmed by varying the pressures and vessel sizes.

## Results

### Device design and testing

**Figure 1:**
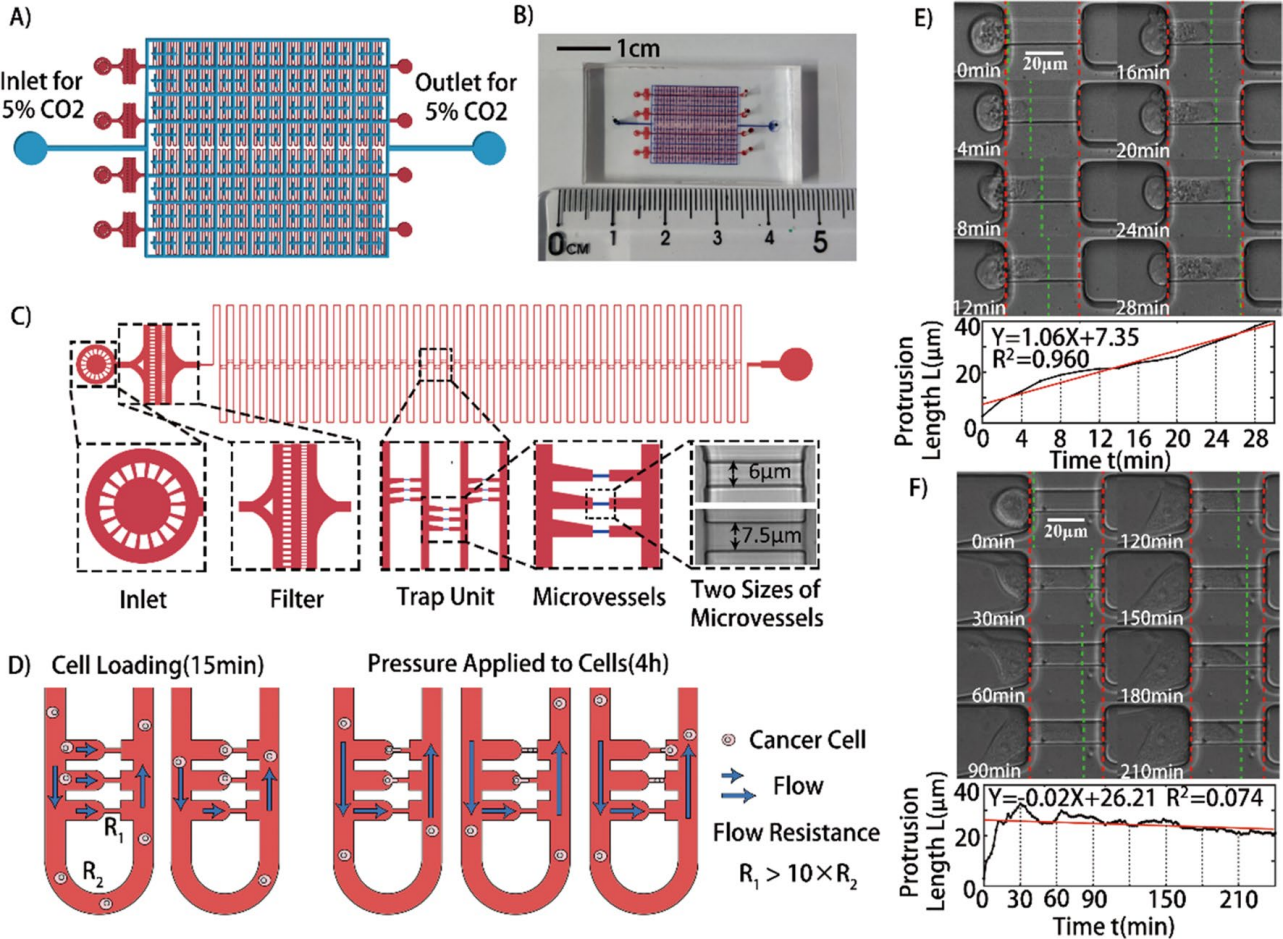
The design principle of the microfluidic device and time-lapse microscopy images of two typical cells with different traverse-vessel behaviors. (**A**) Schematic diagram of the double-layer microfluidic chip with CO_2_ layer (*blue*) and cell cultivation layer (*red*) to maintain cell culture environment and culture cells. (**B**) A photograph of the microfluidic chip with CO_2_ layer (*upper, blue*) and cell cultivation layer (*bottom, red*). Scale bar: 1 cm. (**C**) Detailed schematic diagrams of the cell cultivation layer with Inlet, Filter, Trap Unit and Outlet. (**D**) Schematic illustration of experimental design in the research. (**E**,**F**) Time-lapse microscopy images and the protrusion length as a function of time for two typical cells under applied pressure difference $\Delta {\textit{P}}$, 200 mbar and 100 mbar respectively. Scale bar: 20 μm.

The design concept and a photograph of the whole microfluidic device are shown in Fig. 1A,B. The microfluidic device was manufactured by bonding the CO_2_ layer (upper) on the cell cultivation layer (bottom) using air plasma. Throughout the experiment, 5% CO_2_ was pumped into the upper layer and diffused into the bottom layer. Meanwhile, the whole microfluidic system was heated to 37 °C. These methods could maintain the vitalities of single cells trapped in microvessels.

Specifically, the cell cultivation layer consists of four parallel identical microchannels with inlets, filters, trapping areas and outlets, as detailed in Fig. 1C. The filter structures (20 μm in width) are designed to block cell clusters and large debris. The trapping area is composed of a main square-wave shaped loop channel (40 μm in width, 20 μm in height, 40 loop and 7.2 mm long for each loop) and repeated triplet capillary-like straight channels positioned along both sides of the axis. For microvessels, there were two sizes available for our experiments. The width, height and length of the capillary-like channels were 7.5 μm × 6 μm × 40 μm and 6 μm × 5 μm × 40 μm, respectively, which were comparable to conventional micropipette studies.

The experimental design is shown in Fig. 1D. The flow through the narrow channels will carry single cells into the trap based on the principle of flow resistance^25^. In our design, cells with diameters of less than 16 μm could be captured when they flow on the side of trap units. In addition, the geometric parameters of the microfluidic chip are fine-tuned to ensure that the inequation, ${\textit{R}}_{1}> \text{10} {\textit{R}}_{2}$, is satisfied, where ${\textit{R}}_{1}$ is the flow resistance of narrow straight channels and ${\textit{R}}_{2}$ is the flow resistance of loop channels. Therefore, the pressure differences applied to the cells in the narrow straight channels could be kept the same regardless of whether other narrow straight channels were blocked by cells. After the cell loading period, approximately 15 min in our experiments, sufficient numbers of single cells could be captured. Then, predefined pressures were applied to cells for 4 h, and their dynamic behaviors were recorded using time-lapse microscopy. In our experiments, we applied pressure differences ${\Delta}{\textit{P}}$, 50 mbar, 100 mbar, 200 mbar or 400 mbar, that is, 5.0 × 10^3^ Pa, 1.00 × 10^4^ Pa, 2.00 × 10^4^ Pa and 4.00 × 10^4^ Pa to the four channels in one device, corresponding to pressure drops ${\Delta}{\textit{p}}$, approximately 63 Pa, 125 Pa, 250 Pa or 500 Pa on captured cells. As a comparison, the blood pressure in the human body ranges from 3 to 120 mmHg, that is, 400 Pa to 1.6 × 10^4^ Pa, and the lymph pressure could be as low as 1 mmHg, that is, 133 Pa. To make the logic clearer, it is worth emphasizing that the unit of Pa is used to measure the pressure drops which captured cells felt, the unit of mbar is used to measure the pressure differences which were applied to the whole microfluidic device, and the unit of mmHg is used to measure the blood or lymph pressure throughout the paper.

To ensure the effectiveness of the microfluidic device, preliminary experiments and simulations were performed to test the occupied rate of trap units and estimate the pressure drops on isolated cells. As shown in Fig. S1A, the occupied rate of trap units increases with the cell density during the period of cell loading. As illustrated in Fig. S1B,C, simulations of fluid flow using COMSOL (COMSOL Multiphysics) software indicate that as the pressure difference applied on the whole chip increases geometrically, the pressure drops exerted on single cells increase in a similar manner with an acceptable deviation from defined expected pressure drops. In our experiments, the occupied rate was controlled at approximately 20–50% using a suitable cell density (Fig. S1A). Under these conditions, the design of our microfluidic device could meet the experimental requirements to compare the behaviors of cells under predefined pressure differences in this work.

Under fixed pressure, cells enter the microvessels, clog the flow, and bear the force. The cell width could fill the microvessel width in most cases to guarantee the force exerted on cells, as expected. Using the microfluidic chips demonstrated previously, a large amount of data about the dynamic behaviors of single cells at fixed pressures were recorded. Customized MATLAB codes were used to identify and analyze these dynamic behaviors of the single cells across the microvessels. In our experiments, the protrusion length *L* of a single cell is defined as the length of the trailing edge of the single cell into the microvessels. Focusing on the period that cells enter the microvessels and reach the ends, two kinds of patterns of dynamic behavior were recognized through data analysis, as demonstrated in Fig. 1E,F, through linear fitting (see Movies S1 and S2 for details). One pattern displays excellent linearity (R-squared value approximately 0.96), suggesting the Newtonian droplet state, and the other elucidates that some cells might behave in a complex and nonlinear manner after adhesion with microvessels.

### Dynamic patterns under fixed pressure drops

**Figure 2:**
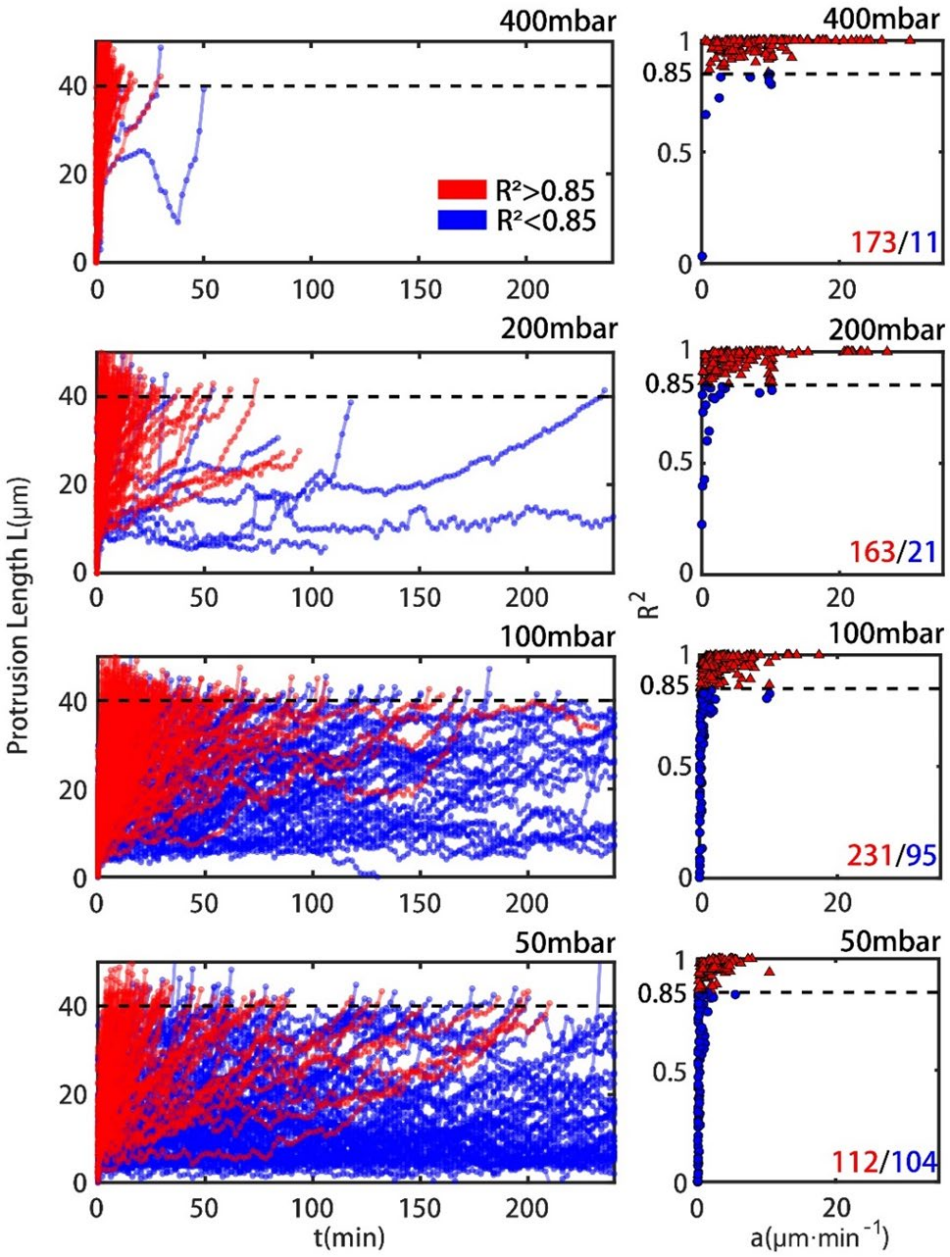
Left: Dynamic behaviors of cancer cells traversing microvessels with 7.5 μm × 6 μm × 40 μm over time under four applied pressure differences $\Delta {\textit{P}}$ (400 mbar, 200 mbar, 100 mbar and 50 mbar); Right: Scatter plot of R-square and fitting velocities for cells traversing microvessels with 7.5 μm × 6 μm × 40 μm under four applied pressure differences $\Delta {\textit{P}}$ (400 mbar, 200 mbar, 100 mbar and 50 mbar). Red lines and Blue lines indicated cells with R-squared of linear fitting larger or smaller than 0.85.

All the dynamic behaviors of cells under all different pressure drops could be observed in our experiments, as depicted in Figs. 2 and S2. Detailed analysis indicates that some of the single cells show excellent linearity, while the rest behave nonlinearly. In previous studies on the responses of single cells under high stresses in seconds, most results have emphasized that single cells should be considered elastic or viscoelastic materials^5,26^. That is, the linear elastic solid model has been widely applied and worked well in this field. Nevertheless, our research demonstrates that viscosity plays a dominant role instead of elasticity under low mechanical stresses for minutes to hours. A classification was performed based on the R-squared value of linear fitting, as demonstrated in Fig. 2. The classification threshold of 0.85 was chosen to discriminate the “linear” cells from the “nonlinear” cells. Obviously, the ratio of the “linear” cell number to the number of total cells decreased from 99 to 48% when the pressure difference decreased from 400 to 50 mbar. More cells exhibit complicated and nonlinear behaviors under a lower pressure drop. The same experiments were carried out not only in the MCF-7 cell line but also in the MDA-MB-231 cell line (Fig. S3).

For most adherent cell types, such as cancer cells, the adhesion time scale ranged from tens of minutes to several hours^27^. The dynamic behaviors of adherent cells can be tuned by related proteins, such as integrin and talin^2,3^. The results seem to indicate that in our experiments, cells are likely to switch from the suspended state to the adherence state. To ensure the existence of cell adhesion, additional immunostaining experiments were performed to display the distribution of vinculin and F-actin in the traverse-vessel behaviors as discussed in the Supplementary Information (Supplementary Figs. S4, S5 and S6). Obviously, the biochemical reactions during cell adhesion and random migration might make a significant contribution to cells’ traverse-vessel behaviors for the “nonlinear” cases.

### Modified Newtonian droplet model

**Figure 3:**
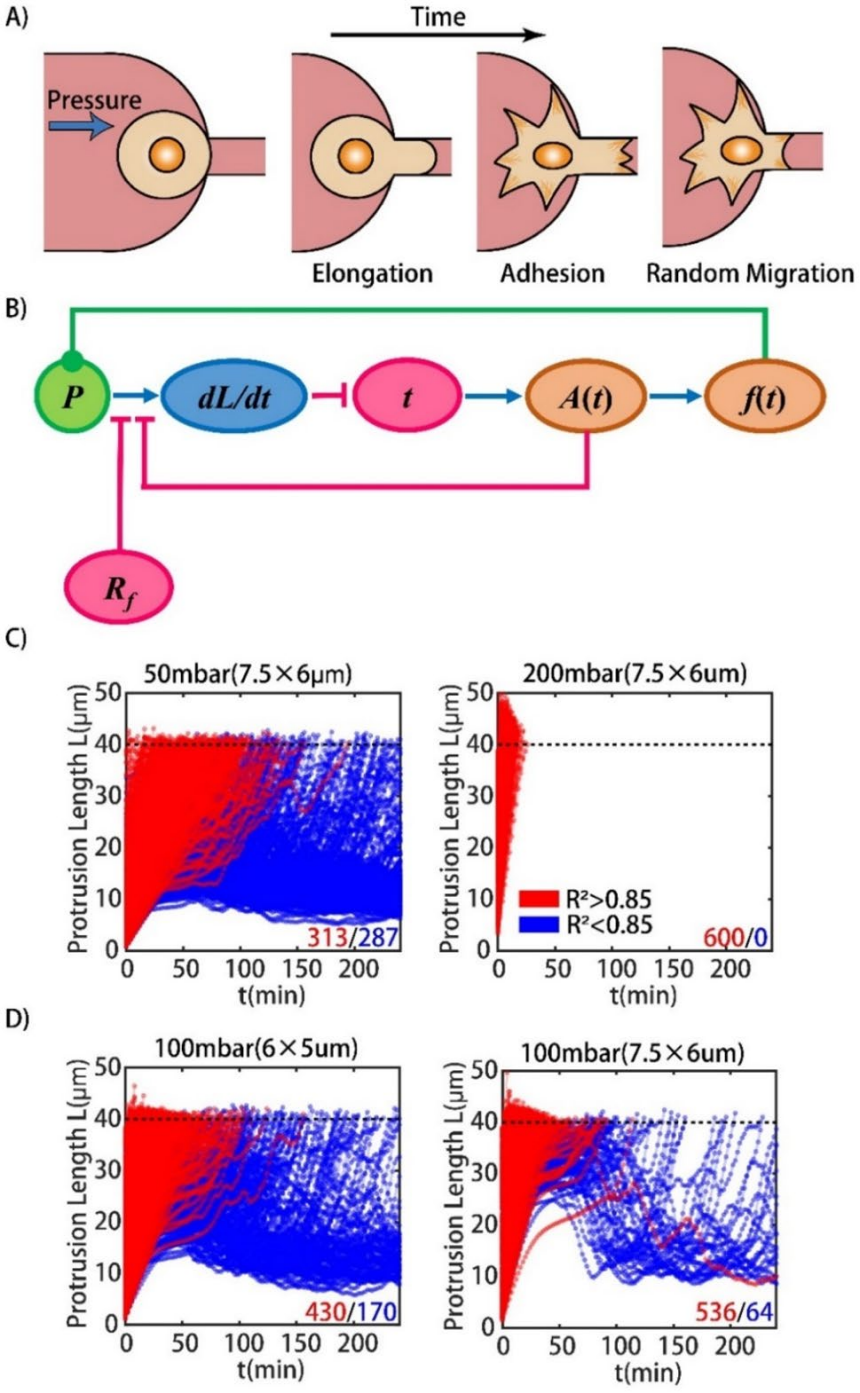
The model and the simulation of traverse-vessel behaviors. (**A**) Sketch map of cell elongate, and adhere/migration in the microvessel. (**B**) The model for the dynamic behavior of cells in the microvessel. (**C**) The simulation of dynamic behaviors for cells under different pressure differences (50 mbar and 200 mbar) using the microfluidic chips with the same size of microvessels (7.5 μm × 6 μm). (**D**) The simulation of dynamic behaviors for cells under the same pressure difference (100 mbar) using the microfluidic chips with different sizes of microvessels (6 μm × 5 μm and 7.5 μm × 6 μm).

The basic Newtonian droplet model regards a single cell as a Newtonian droplet^26^, and the behavior of these cells can be elucidated using a linear differential equation. This means that the viscosities of these “linear” cells remain constant during the whole dynamic process. This model can only explain the linear behaviors of single cells and has no explanatory ability for the other “nonlinear” cells. As shown in Fig. 3A, these “nonlinear” cells elongate in the microvessels and may adhere to the microvessels with time; then, they behave more randomly.

To describe these complicated and nonlinear dynamic patterns, we illustrated the dynamic behavior mechanism, as shown in Fig. 3B, and modified the original Newtonian droplet model (details given in the Supplementary Information):

1 $$\frac{dL}{{dt}} = \frac{{\Delta p \cdot S - R_{f} }}{{6\eta_{int} \pi r}}$$

2 $$\frac{dL}{{dt}} = \frac{{\Delta p \cdot S - R_{f} + f(t)g(L)}}{{6\eta_{int} \pi r + \alpha A(t)}}$$

${\textit{L}}$ is the protrusion length of the cell into the microvessel, ${\Delta}{\textit{p}}$ is the applied pressure to the cell, ${\textit{S}}$ is the area of the cross-section of the microvessel, *R*_*f*_ is the resistance corresponding to the critical excess suction pressure because of the capillary effect, *η*_*int*_ is the intrinsic viscosity of the cell, and $r$ is the equivalent radius of the cross section of the microvessel.

In the modified model, *α·A*(*t*) is introduced into the formula according to the cell adherence effect and may greatly contribute to overcoming the pressure difference, where *A*(*t*) is defined as the degree of cell adhesion over time (Supplementary Fig. S7), and *α* is the constant coefficient between cell adhesion and additional viscosity. *f*(*t*)*·g*(*L*) is used to describe the active force generation during the whole traverse-vessel behavior, where *f*(*t*) is the value of the active force, and *g*(*L*) is a tunable factor. Here, we assumed that the active forces *f*(*t*) generated by cells are distributed randomly in the allowable range from –*A*(*t*) to *A*(*t*). *g*(*L*) depicts the effect of the protrusion length on active force generation. The simulation parameters were determined based on previous studies (details given in Supplementary Table S1).

As shown in Fig. 3C, these simulation results are mostly in agreement with our experiments. Under a low pressure drop, cells behave like Newtonian droplets before adhesion, and the nonlinearity of cells becomes more pronounced during adhesion. Most cells behave in an excellent linear manner under a high pressure drop. That is, the active force generated by biochemical reactions has a prominent influence on cell behaviors during the traverse-vessel process. To confirm our conjectures, this parameterized model was used to simulate cell behaviors in narrower microvessels. As expected, Fig. 3D illustrates that more cells behave “nonlinearly” in narrower microvessels under the same pressure drop. Detailed data analyses demonstrated that our model could explain most experimental phenomena for two sizes of microvessels (Supplementary Figs. S8, S9).

### Distribution analysis of cell behaviors illustrated mode switching

As discussed in the model and Eqs. (1) and (2), the cells may act like a Newton droplet phase and transform to an adhesion/migration phase with increasing contact time. In addition to the R-squared value, we also used apparent viscosity (*η*_*app*_) to indicate the cell behavior changes. The apparent viscosity (*η*_*app*_) is defined as:

3 $$\eta_{app} = \frac{{\Delta p \cdot S - R_{f} }}{{6\pi r\dot{L}}}$$

**Figure 4:**
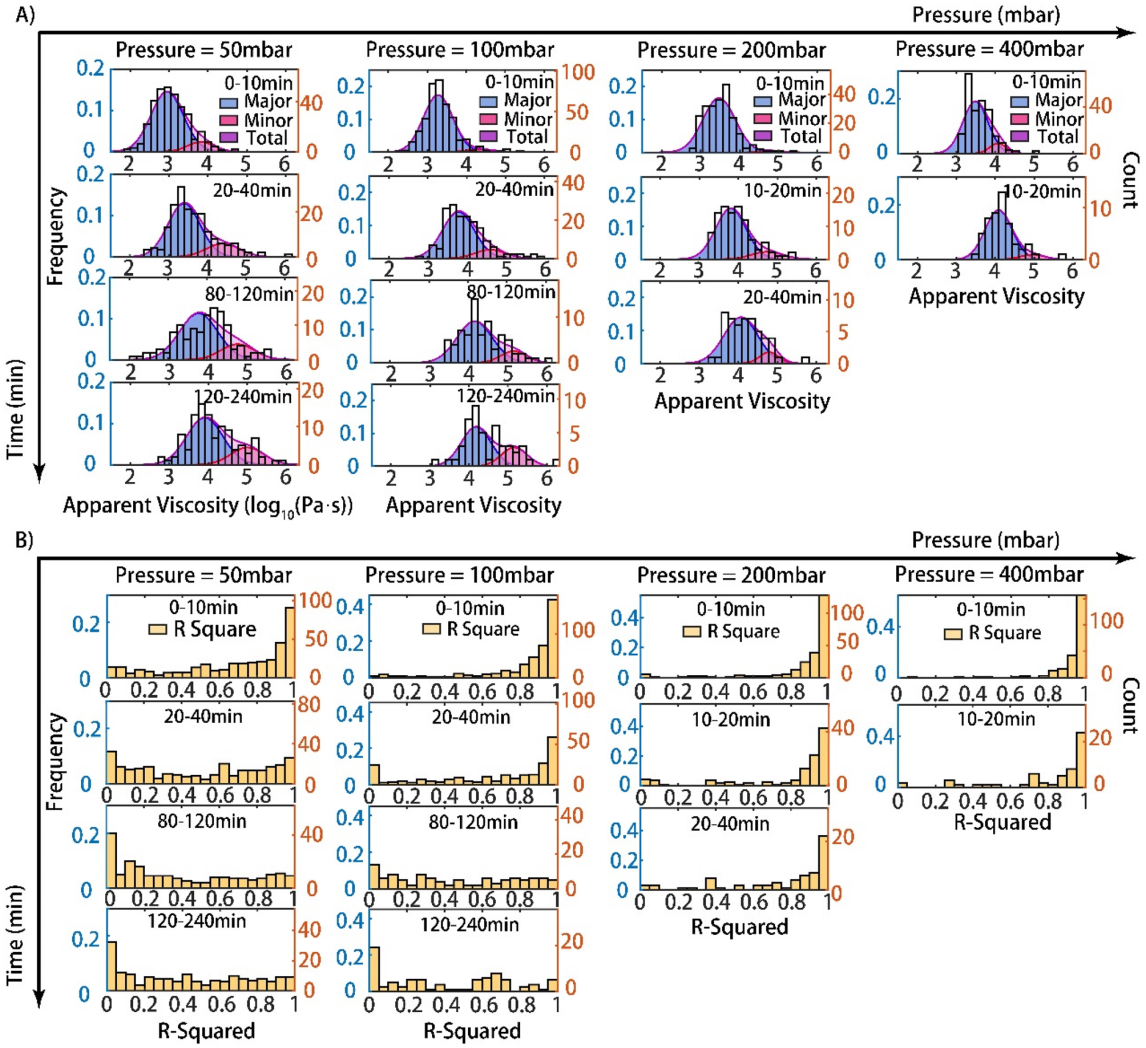
Distribution analysis of cells’ behaviors illustrated the transition. (**A**) Probability density distribution of cell apparent viscosities for 50, 100, and 200 and 400 mbar at various periods (0–10 min, 20–40 min, 80–120 min, 120–240 min). Major peak in blue, minor peak in pink and total in purple. (**B**) Probability density distribution of the R-squared of linear fitting for 50, 100, and 200 and 400 mbar at various times. The left y-axis shows frequencies and the right y-axis gives counts for both (**A**) and (**B**).

As shown in Fig. 4A, under a constant pressure drop, the major peak (*blue*) shifts toward a higher apparent viscosity, indicating that the apparent viscosity of the cells gradually rises. Meanwhile, a weak peak (*pink*) at greater apparent viscosity occurs and increases in the proportion of total viscosity over time. This behavior is more remarkable under a lower constant pressure drop, while under a higher pressure drop, cells often traverse through the capillary before they can adhere to their surrounding environment. The R-squared distribution in Fig. 4B could also offer support for this conjecture. Under the lower consistent pressure drop, cells mostly showed a relatively great linear behavior within dozens of minutes, and then cells progressively transitioned from a Newtonian droplet state to an adhesion/migration state. Finally, cells completed the transitions and exhibited nonlinear behaviors. All the distributions of apparent viscosity and R-squared values within four hours can be found in Supplementary Fig. S10 (experiment) and Fig. S11 (simulation).

**Figure 5:**
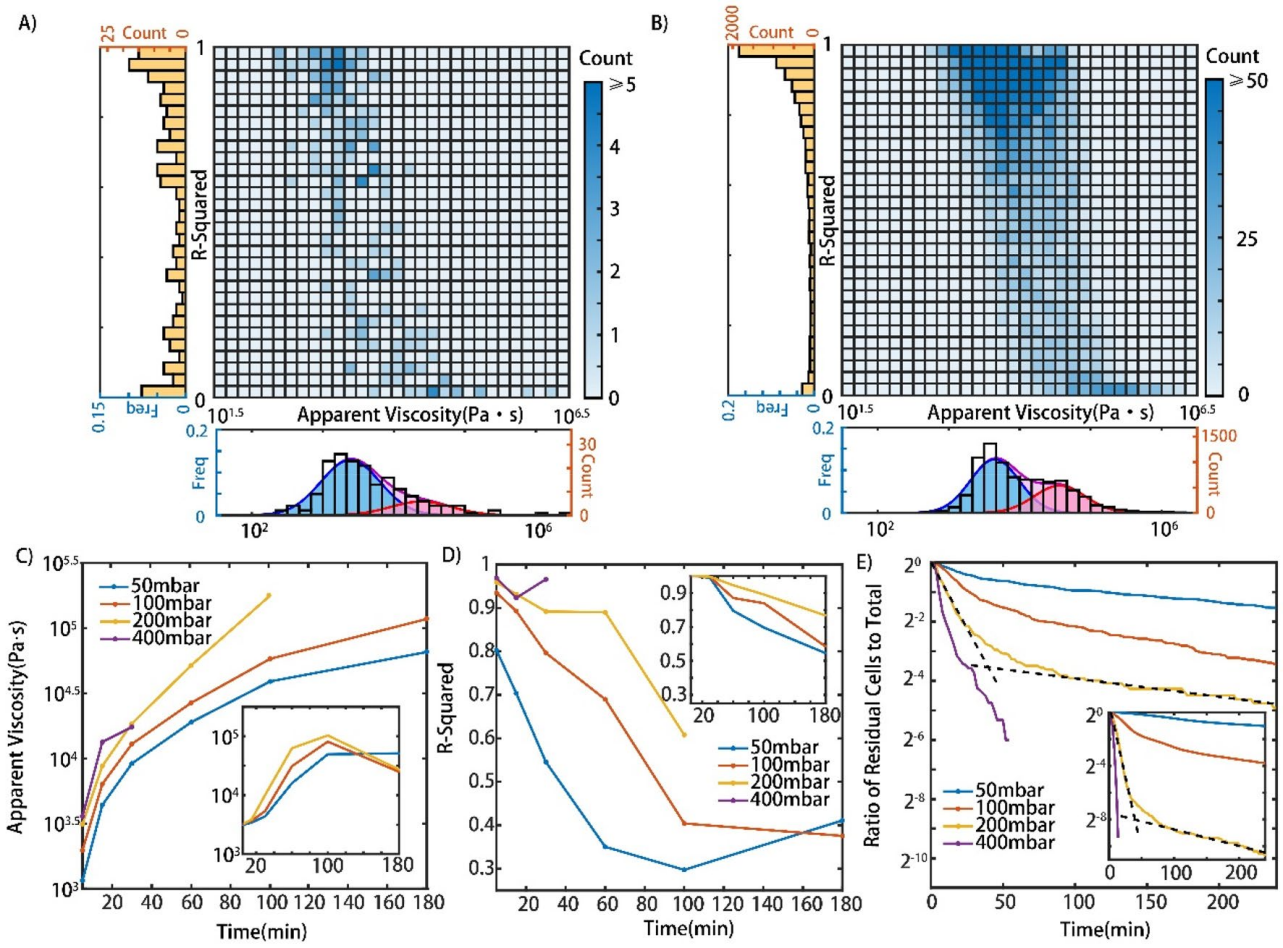
(**A**,**B**) Heatmap depicting the number of cells with corresponding R-squared and apparent viscosities under the pressure difference 50 mbar. (**A**) Counts cells between 20 and 40 min in experiments. (**B**) Counts cells between 40 and 80 min in simulations. (**C**,**D**) Medians of apparent viscosities and R-squared of cells (up to 400 cells in the first time frame under the pressure difference 50 mbar, no less than 20 cells in the final time frame under the pressure difference 400 mbar) over time under different pressure differences in experiments. Insets are in simulations. (**E**) Ratio of residual cells (larger than 200 cells in any condition at the start of experiments) to total throughout the experiments under different pressure differences. Inset is in simulations. Dashed lines represent results of linear fitting for two distinct stages.

To further confirm the transition and compare experiments with theories, the numbers of cells with specific R-squared values and apparent viscosities in experiments and simulations are illustrated in Fig. 5A,B. It is worth noting that Fig. 5A counts cells between 20 and 40 min and Fig. 5B counts cells between 40 and 80 min both under a pressure difference of 50 mbar. These results indicate a negative correlation between the apparent viscosities of single cells and R-squared values regardless of experiments or simulations. In addition, the heatmaps also show two evident clusters in the upper left and the lower right, suggesting the existence of two potentially distinct states for cells. One state had lower apparent viscosities and higher R-squared values close to 1, and the other had higher apparent viscosities and lower R-squared values close to 0. To eliminate doubt about whether the number of single cells is enough to prove this conjecture, a heatmap depicting the accumulation of the numbers of cells during different periods is shown in Fig. S12. The comparison of Fig. S12A,F could elucidate the transition between these two states over time. Similar results are also observed in simulations, as shown in Fig. S13. Taken together, these results confirm that cancer cells can switch their state from a Newtonian droplet state to an adhesion/migration state.

Throughout these transitions, the apparent viscosities of cells increased initially and then leveled off with time, indicating that cells attached to the surrounding environments completely, as shown in Fig. 5C. Meanwhile, Fig. 5D suggests that the dynamic behaviors of cells are influenced by cell adhesion and become more nonlinear than before. Finally, considering the residual cells before traversing the microvessels, the ratio of these cells to the total number of cells implies two potential distinct stages, a rapid decline at the initial stage and a steady decline at the latter stage, as plotted in Fig. 5E using dotted lines, which also suggests the mode switch. From the beginning to the end, biochemical reactions initially have little effect but dominate these transitions. Experiments and simulations both verified these phenomena qualitatively, although they might not match perfectly from a quantitative view because the elapsed time of cell loading cannot be guaranteed to be the same in all experiments, and the parameters chosen in simulations might not be best suited for all experiments.

### Further discussions of the effects of pressure and vessel size on cell behaviors

**Figure 6:**
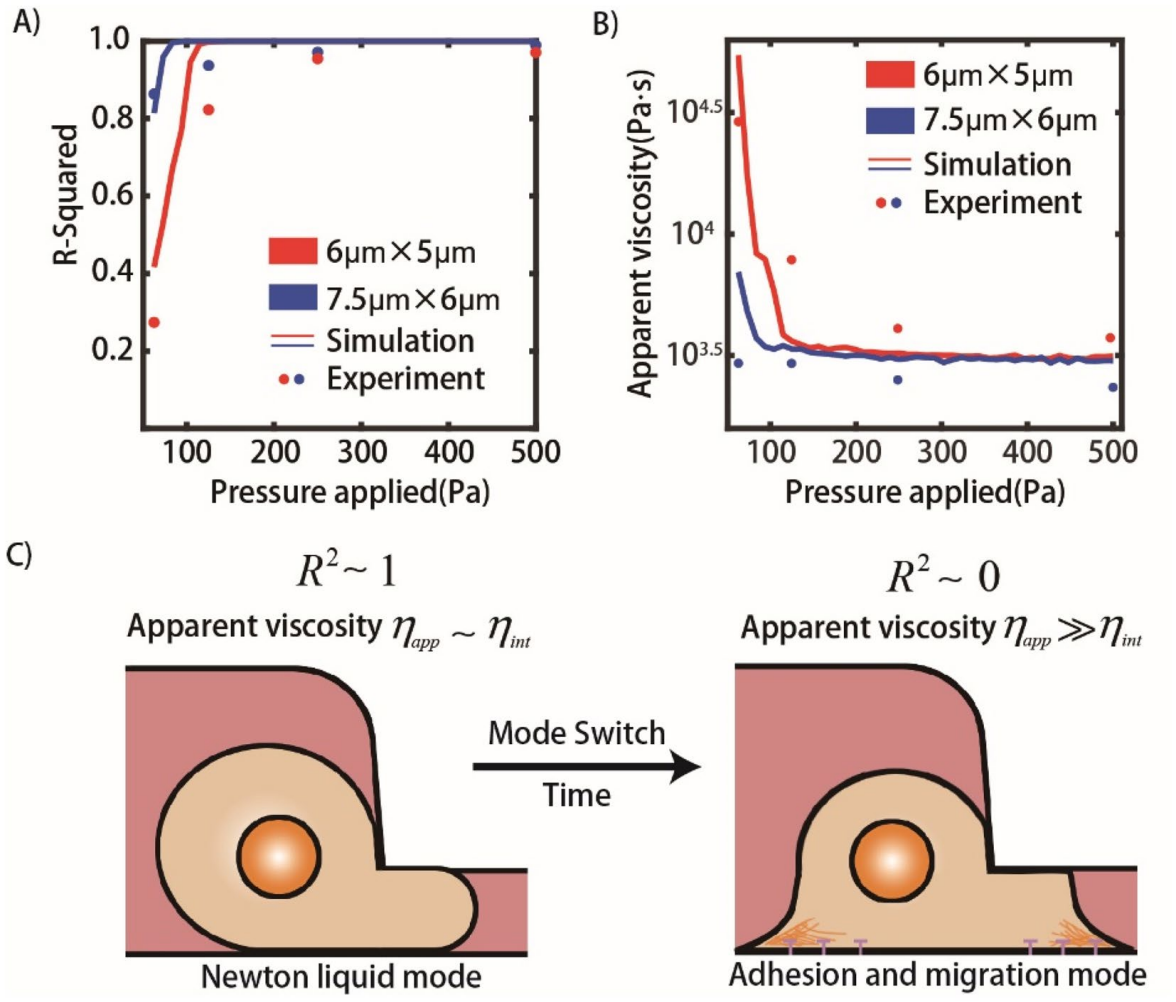
The summarization of the R-squared and the apparent cell viscosity of the cell traverse-vessel behaviors at different conditions. (**A**,**B**) The comparison of plots of R-squared and cell apparent viscosity as functions of pressure applied for experiments and simulations under different pressure differences (median of all available cells in one condition, and no less than 80 cells in any condition at any time). (**C**) Illustration of cell transition from Newtonian liquid mode to adhesion and migration mode.

The dynamic behavior of cells in microvessels of different sizes under different pressures is summarized and compared in Fig. 6 to further verify our parameterized model.

To compare the R-squared values and apparent cell viscosities of the cell traverse-vessel behaviors under different conditions in experiments with simulations, simulations were performed densely under a pressure drop ranging from 63 to 500 Pa. Figure 6A shows the median value of the fitted R-squared values of all cell behaviors with different pressure drops. As the pressure drops decreased, the R-squared value decreased from approximately 1.0 to a relatively low value, ~ 0.8 for microvessels with 7.5 μm × 6 μm × 40 μm and ~ 0.4 for microvessels with 6 μm × 5 μm × 40 μm. The smaller the microvessel size was, the larger the decrease in the median R-squared value. In contrast, Fig. 6B demonstrates that the median of the apparent viscosities *η*_*app*_ of cells increases greatly from ~ 3000 Pa·s as the applied pressure decreases. The value of cell apparent viscosities approximately matches previous studies^28–30^. The experimental results are in good agreement with the simulation results in Fig. 6A,B.

To illustrate the core concept, schematics of two kinds of dynamic patterns are depicted in Fig. 6C. Before adhesion, cells could linearly traverse the capillary-like vessels, and their apparent viscosities *η*_*app*_ are approximate to the real viscosities *η*_*int*_ of these cells. Conversely, if pressure is too low or the microvessel is much smaller, the cells would adhere to the microvessels and generate active force to speed up or slow down their migration. After adhesion, these cells behave randomly and nonlinearly, and then the apparent viscosities *η*_*app*_ of these cells are much larger than their real viscosities *η*_*int*_. In this state, these cells could maintain their physiological conditions and seed in current sites, which means that cells might become more aggressive and form a metastatic tumor. It is worth mentioning that the adhesion effect is much more important than the random factor of f(t), which causes most of the changes in apparent viscosities and R-squared value calculated for cells traversing microvessels (Fig. S14). In other words, if we could reduce the adhering force between cancer cells and microvessels, cancer metastasis might be restrained to some degree. By using PLL-g-PEG to coat the channel surface, which decreased the adherence effect of cancer cells, we found that the traversing behaviors were more linear and that the apparent viscosities of the cells were smaller than those of the uncoated condition (Fig. S15).

## Summary and discussion

We designed a high-throughput microfluidic system with an external pneumatic pump to research the dynamic behaviors of cancer cells over long timescales responding to low mechanical stresses. We have demonstrated that cancer cells might behave in two quite different patterns, “linear” and “nonlinear”, when they exert a low pressure drop, and this transition from “linear” to “nonlinear” is continuous as the applied pressure decreases from 500 to 63 Pa. Using the R-squared value of linear fitting as the evaluation criterion, the ratio of the “nonlinear” cell number to the number of total cells increases during the reduction of applied pressure. To explain these phenomena, we modified the Newtonian droplet model and introduced the influence of biochemical reactions that occur in cell adhesion and random migration. In addition, simulations and experiments could match well regardless of different sizes of microvessels and different applied pressures. Generally, we verified how the size of vessels and the pressure differences affected cancer cells’ traverse-vessel behaviors. We believe that our research could provide some useful insights for cancer metastasis and cancer therapy.

## Materials and methods

### Cell culture

Metastatic human breast cancer cells (MCF-7 and MDA-MB-231 cell lines) used in the test were purchased from American Type Culture Collection (ATCC). Cells were cultured in Dulbecco’s modified Eagle’s medium (DMEM; Gibco) supplemented with 10% fetal bovine serum (FBS; HyClone) and 100 U/mL penicillin and streptomycin (Gibco) at 37 °C in 5% CO_2_.

### Device fabrication

Photolithography was used to fabricate the master molds. Briefly, mold 1 of layer 1 for cell loading and pressure operation was fabricated by SU8 (MicroChem, USA) with two heights. Then, the mixture (A:B = 8:1) of prepolymer polydimethylsiloxane (PDMS) was spin-coated on the mold of layer 1 with a height of approximately 50 μm and cross-linked at 70 °C for 2 h. Mold 2 of layer 2 for a 5% CO_2_ supply was also fabricated by SU8 with a height of 40 μm. A mixture (A:B = 12:1) of PDMS was cast on the mold of layer 2 with a height of approximately 8 mm and cross-linked at 70 °C for 2 h. Then, the PDMS layer on mold 2 was peeled off and bonded to layer 1 by air plasma, as shown in Fig. 1A. After peeling off the bonded PDMS piece from mold 1 and punching the inlets and outlets, the PDMS piece was bonded to the glass slide by air plasma. Once the PDMS chip was sealed against the glass slide, the device was placed in an oven at 70 °C overnight to improve bonding quality before use.

### Experiments and analysis

Before the experiments, the device was exposed to UV and degassed in vacuum for 30 min for sterilization. We used 0.25% trypsin–EDTA solution to dissociate MCF-7 cells at 37 °C for 3 min. These cells were resuspended to a concentration of 10^5^/mL in normal medium. Cells were loaded into the device through the inlets and trapped into capillary-shaped microvessels for 15 min under fixed pressure (50 mbar). Then, the sample positions were picked up under a Nikon Ti microscope with a standard dry objective (Nikon LWD 40×/0.55 Ph2 ADL) after stopping the air-pressure pump to ensure the recovery of trapped cells (approximately 10 min). After setting different pressures for different channels by a four-channel pressure operator (Fluidic controller, MesoBiosystem, China), the microscope system captured images at 2 min per frame in the regions of interest. The zoom knob was set to 1.5× for more magnification. Experiments typically lasted 4 h. Experimental analysis and cell tracking were performed using custom MATLAB programs.

## Supplementary Information

Supplementary Information 1.

Supplementary Video 1.

Supplementary Video 2.

## Data Availability

All data generated or analyzed during this study are included in this article and its supplementary information files. These datasets are also available from the corresponding author on reasonable request.

## Code availability

The MATLAB codes in the current study are available from the corresponding author on reasonable request.
