# Supplementary material for: Biophysical studies of cancer cells’ traverse-vessel behaviors under different pressures revealed cells’ motion state transition
Source: Sci Rep. 2022 May 5;12:7392. doi: 10.1038/s41598-022-11047-5 (PMC9072532; doi:10.1038/s41598-022-11047-5)
Supplement: Supplementary file 1 — Supplementary Information 1. [file 41598_2022_11047_MOESM1_ESM.docx]

**Supplementary Information for**

Biophysical studies of cancer cells’ traverse-vessel behaviors under different pressures revealed cells’ motion state transition

**Xiao Li**^1,2^**, Jialin Shi**^1^**, Ziqing Gao**^3^**, Jian Xu**^1^**, Shujing Wang**^1,2^**, Xin Li**^2^**, Qi Ouyang**^1,2,3^**, Chunxiong Luo**^1,2,4,5^*****

^1^The State Key Laboratory for Artificial Microstructures and Mesoscopic Physics, School of Physics, Peking University, China.

^2^Center for Quantitative Biology, Academy for Advanced Interdisciplinary Studies, Peking University, China.

^3^Peking-Tsinghua Center for Life Sciences, Peking University, Beijing, China.

^4^Wenzhou Institute University of Chinese Academy of Sciences, Wenzhou, Zhejiang, China.

^5^Oujiang Laboratory, Wenzhou, Zhejiang, China.

*****To whom correspondence should be addressed: Chunxiong Luo, pkuluocx@pku.edu.cn.

**Supplementary Information Text**

**S1 Device design and testing**

Fig. S1A illustrates that the microfluidic devices could capture single cells as designed and that the occupied rate of trap units increases with the cell density during the period of cell loading. To obtain a more precise estimation, we simulated the fluid flow of the microfluidic device and the pressure drops on single cells using COMSOL (COMSOL Multiphysics) software. In Fig. S1B, as the pressure difference applied on the whole chip increases geometrically, the pressure drops exerted on single cells increase in a similar manner despite the occupied rate varying from 20% to 50%. In more detail, for the influence of the occupied rate on the pressure drops, the three-dimensional chip model and an image of the velocity field under an applied pressure difference of 50 mbar are illuminated in Fig. S1C. It is worth noting that the pressure drops on the single cells vary little with different occupation situations of their trap units (65-85 Pa for 1/3 to full occupation).

**S2 Direct evidence of cell adhesion in the traverse-vessel behaviors**

Generally, cells adhere to untreated surface after seeding for 1-2 hour [1-2]. But the adhesion to the PLL-g-PEG coated surface needs far longer than 2 hours [2]. According to the previous studies [3-8], many types of cells could attach to the glass surface, including both the MCF-7 and MDA-MB-231. However, many researchers had reported that the cells could only adhere to PDMS without any special treatment weakly [9-10]. We performed the additional experiments to verify these conclusions and the similar results were observed in our experiments.

In these additional experiments, we set 50 mbar as a fixed variable to ensure the cancer cells would deform their morphologies in the microfluidic channels but not traverse the vessels quickly in the given time. After the pressure was applied for 30 min, 60 min or 120 min, the cells were fixed using 4% paraformaldehyde for 20 min and permeabilized with PBS containing 0.1% Triton X-100 for 3 min in the microfluidic chip.

Then, the cells were incubated in the diluted primary antibody (Anti-Vinculin, EMD Millipore, Lot: 3549975) in 1% BSA for 1 hour. After washing the cells with 0.05% Tween-20, the immunostaining experiments were performed using FITC-secondary antibody (SIGMA, Lot: SLBZ1892) for anti-vinculin and TRITC-Phalloidin (EMD Millipore, Lot: 3381559) for F-actin in the chip. The same experimental operations were also performed on cancer cells cultured on i) commercial polystyrene petri dishes as control, ii) native unmodified PDMS-covered dishes and iii) PDMS-covered dishes which the culture medium was added into and further incubated overnight for comparison.

We recorded the fluorescence images of the cells using spinning-disk confocal microscopy (Andor Dragonfly). The images of cancer cells under the given pressure 50 mbar for 30 min revealed that vinculin was distributed randomly on the cell surface in contact with the glass bottom at the early stage (Fig.S4A). However, after applying the pressure for 60 min or 120 min, as shown in Fig. S4B and S4C, we observed that vinculin was distributed along the edges of cancer cells on the glass bottom. This phenomenon was consistent with the fluorescence images of cancer cells on the commercial polystyrene petri dish. In addition, the structure of F-actin (red) could also be seen clearly in our experiments.

On the native unmodified PDMS-covered dishes, cancer cells only adhered to the PDMS surface weakly as expected when cell density was large (Fig. S5B). On the contrast, for the PDMS-covered dishes which the culture medium was added into and incubated overnight, there was no obvious difference with the cell adhesion on commercial polystyrene petri dish (Fig.S5A and S5C).

Our results also proved that the cell adhesion in the microfluidic chips was mainly on the glass bottom and little on the PDMS surface as shown in the reconstructed 3D image of 120 min pressure-applied sample (Fig.S6).

**S3 Modified Newtonian droplet model**

Yeung and Evans derived the Newtonian droplet model of suspended cells to simulate the dynamic behaviors of cells in the micropipette [11-12]. The cell cytoplasm is regarded as a Newtonian viscous liquid, and the cell cortex is modeled as a fluid layer with constant tension. The specific version of this model for micropipette aspiration experiments is described by:

(S1)

where ∆*P* is the applied pressure to the cell, *P*_cr_ is the critical excess suction pressure, *R_P_* is the radius of the micropipette, *R_c_* is the radius of the cell body outside the micropipette, *η* is the viscosity of the cell, and *m* is a coefficient that depends on the features of cell structures; for most cases, *m*≈6*.* This model is used to explain the dynamic phenomenon of cells under pretty high suction pressures. It does not suit our experiments well, and some modifications of this model are needed to simulate cells exposed to far lower suction pressures. In our experiments, by setting *R_P_*/*R_c_* ≪ 1, $S=\pi R_{P}^{2}$, could be derived using Eq. (1):

$\frac{dL}{dt}=\frac{\Delta p\cdot S-R_{f}}{6\eta_{int}\pi r}$ (S2)

where *L* is the protrusion length of the cell into the microvessel, Δ*p* is the applied pressure difference on a single cell, *S* is the area of the cross-section of the microvessel, *R_f_* is the resistance corresponding to the critical excess suction pressure because of the capillary effect, *η_int_* is the intrinsic viscosity of the cell, and *r* is the equivalent radius of the cross-section of the microvessel.

**S3.1 Assumptions of the model**

According to previous studies, we make a few assumptions to modify the Newtonian droplet model:

1) The great diversity of cancer cells inevitably leads to the high statistical dispersion of their mechanical properties. In this model, we simplify this situation and assume that the cell intrinsic viscosities *η_int_* are expected to obey the normal distribution with mean *μ* and variance *σ*^2^. This feature will not change over time:

$\eta_{\text{int}}\text{\textasciitilde}\text{N}\text{(}\text{μ}\text{, }\text{σ}^{2}\text{)}$ (S3)

In addition, to describe potential changes in cell viscosities during our experiments, we introduce cell apparent viscosities *η_app_* into the model, which can be calculated through the following equation:

(S4)

For convenience, we use the average speed of cellular protrusion during the whole dynamic process to estimate the apparent viscosity. That is, if no other factors interfere, the equation *η_app_* = *η_int_* can be derived through Equation (S4).

2) During experiments, cells attached to the material surface over time. In this model, we assume that this entire dynamic process of cell attachment, *A*(*t*), could be described using a logistic curve, as previous studies illuminated [13-15]. It is obvious that the curve profile depends on the cell type and surrounding environments shown in Fig. S7A. In our simulations, the cancer cell adhesion strength gradually increased according to the following equation:

$A(t)=\frac{F_{\max}-F_{\min}}{1+e^{k_{1}-t/k_{2}}}+F_{\min}$ (S5)

where *k*_1_ and *k*_2_ could be determined using the beginning and end of cell adhesion, *T*_Ad0_ and *T*_Ad1_, which satisfy the following equations:

$\text{A}\text{(}\text{T}_{\text{Ad0}}\text{) = }\text{F}_{\text{min}}\text{ + 0.01·(}\text{F}_{\text{max}}-\text{F}_{\text{min}}\text{)}$ (S6)

$\text{A}\text{(}\text{T}_{\text{Ad1}}\text{) = }\text{F}_{\text{min}}\text{ + 0.99·(}\text{F}_{\text{max}}-\text{F}_{\text{min}}\text{)}$ (S7)

3) The apparent cell viscosities increase with the adhesion strength *A*(*t*). When cells adhere to the material surface completely, the cell apparent viscosities *η_app_* should reach their maximum and be much greater than the cell intrinsic viscosities:

*η_app_* ≫ *η_int_* (S8)

4) Traction forces *f*(*t*) exerted by adherent cancer cells are coupled with the mechanical properties of cells and the interaction with the surrounding environment. In our model, cells generate these forces in random directions after cell attachment. The value of these forces *f*(*t*) is tuned by the protrusion length of cells *g*(*L*), as shown in Fig. S7B, and we assume that the change per unit time of the forces is limited to 1/*n* of the strength of cell attachment *A*(*t*):

$\left|\Delta f(t)\right|\leq\frac{1}{n}A(t)$ (S9)

Given all assumptions above all, Eq. (S2) could be modified as:

$\frac{dL}{dt}=\frac{\Delta p\cdot S-R_{f}+f(t)g(L)}{6\eta_{int}\pi r+\alpha A(t)}$ (S10)

where *α* is the constant coefficient between cell adhesion and extra viscosity. The apparent viscosity is still calculated through Equation (S4), and it is obvious that *η_app_* ≫ *η_int_* under these conditions.

**S3.2 Parameters of the model**

Many previous studies in this field could be referenced to determine the range of parameters in the model as shown in Table S1.

**S4 Distribution analysis of cell behaviors illustrating mode switching**

In our experiments, the probability density distribution of apparent viscosities in the early stages seemed to follow a Gaussian distribution closely on a logarithmic scale. This inspired us to fit the probability density distribution of cell apparent viscosities using a Gaussian mixture model with two components. Specifically, we fitted the rising edge of the probability density distribution and determined the parameters of the left peak (i.e., the major peak), then subtracted the left peak from the probability density distribution and fitted the residual of probability density distributions to find the appropriate parameters of the right peak (i.e., the minor peak). All distribution fitting was completed using MATLAB.


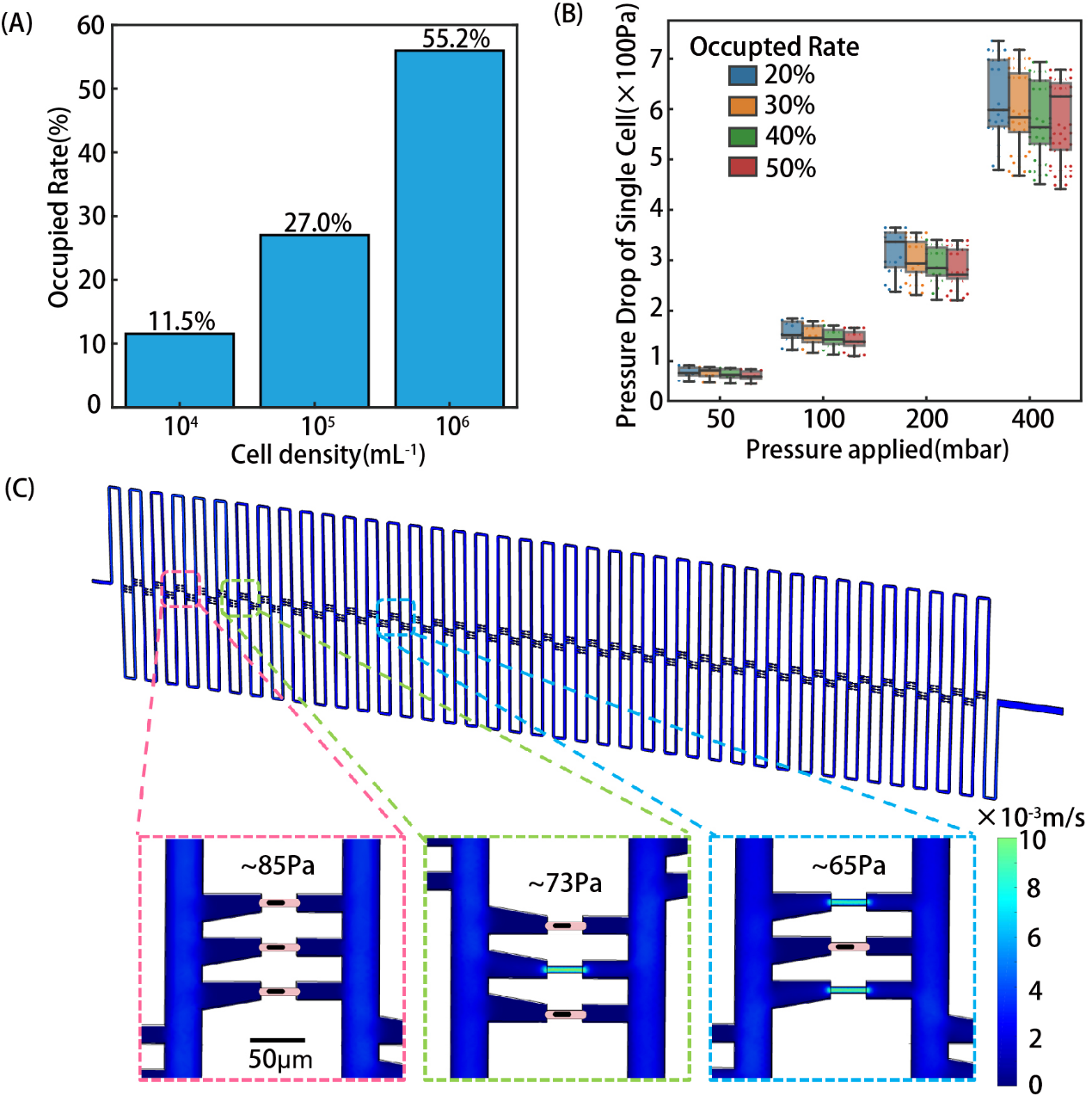


**Fig. S1.** (A) Percentage of cell occupied rate with increased cell densities. (B) The simulation results of pressure drops on single cells at different cell occupied rates under different applied pressure differences. (C) Simulation of the velocity field under an applied pressure difference of 50 mbar. The enlarged views of three trap units indicate that different amounts of captured cells could influence the pressure drop of a single cell.


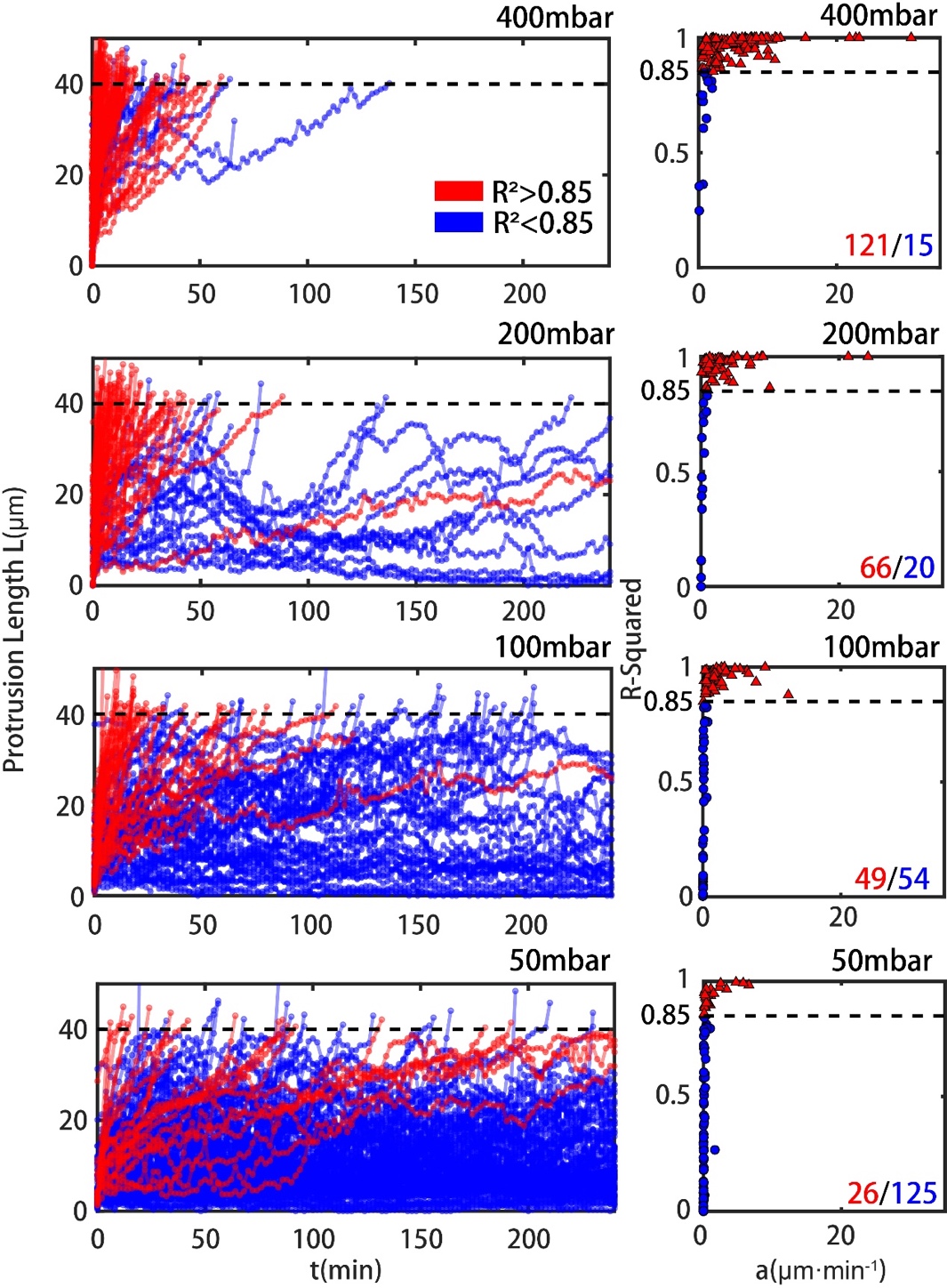


**Fig. S2.** Left: Dynamic behaviors of cancer cells traversing microvessels with 6 μm × 5 μm × 40 μm over time under four applied pressure differences $\text{Δ}\text{P}$ (400 mbar, 200 mbar, 100 mbar, and 50 mbar); Right: Scatter plot of R-squared values and fitting velocities for cells traversing microvessels with 6 μm × 5 μm × 40 μm under four applied pressure differences $\text{Δ}\text{P}$ (400 mbar, 200 mbar, 100 mbar and 50 mbar). Red and blue lines indicate cells with R-squared values of linear fitting larger or smaller than 0.85.

**
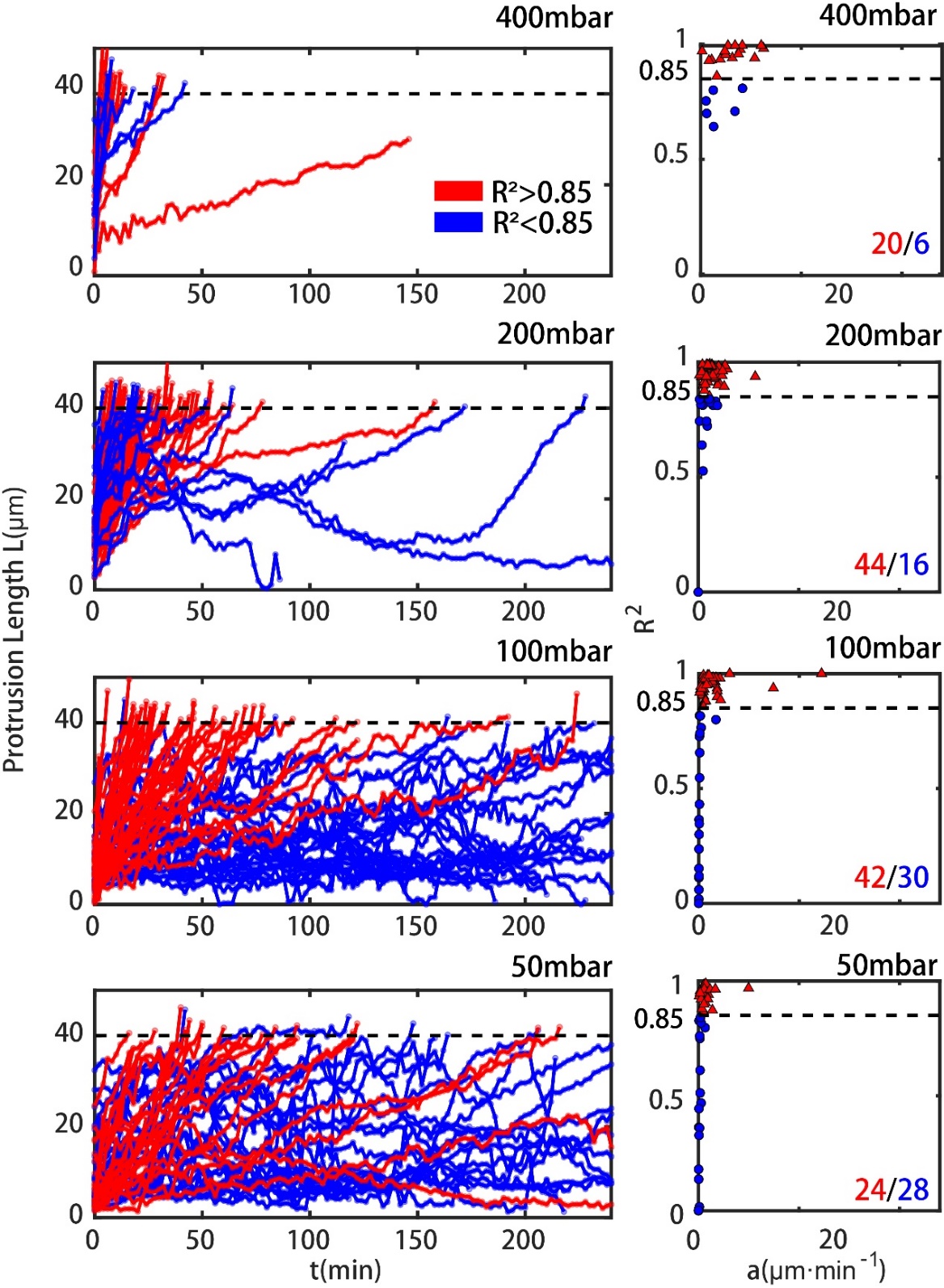
**

**Fig. S3.** Dynamic behaviors of MDA-MB-231 cancer cells traversing microvessels at 7.5 μm × 6 μm × 40 μm over time under four applied pressure differences $\text{Δ}\text{P}$ (400 mbar, 200 mbar, 100 mbar, and 50 mbar). MDA-MB-231 cells show similar behavior to the MCF-7 cell line, which means that the traversing behavior of cancer cells may be universal.


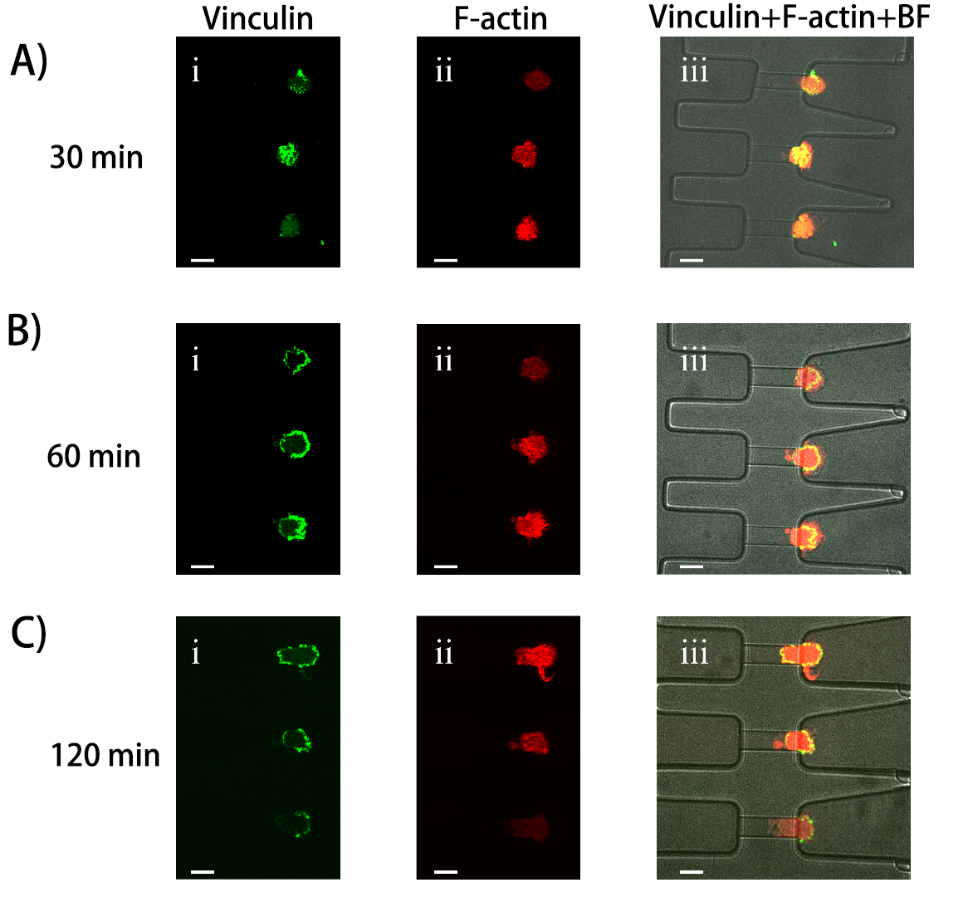


**Fig. S4.** Spinning-disk confocal images of the cancer cells after applying pressure for (A) 30 min (B) 60 min and (C) 120 min. Immunostaining fluorescence images contain (i) FITC-vinculin, (ii) TRITC-F-actin and (iii) merged images. Scalebars: 10 μm.


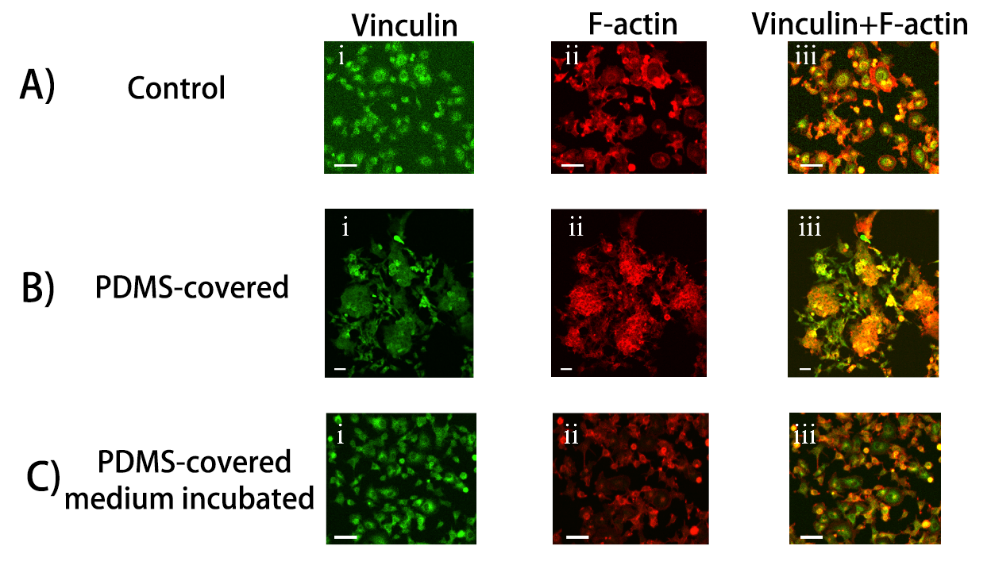


**Fig. S5.** Spinning-disk confocal images of the cancer cells cultured on (A) commercial polystyrene petri dish; (B) native unmodified PDMS-covered dishes; and (C) PDMS-covered dishes which the culture medium was added into and incubated overnight. Immunostaining fluorescence images contain (i) FITC-vinculin, (ii) TRITC-F-actin and (iii) merged images. Scalebars: 50 μm.


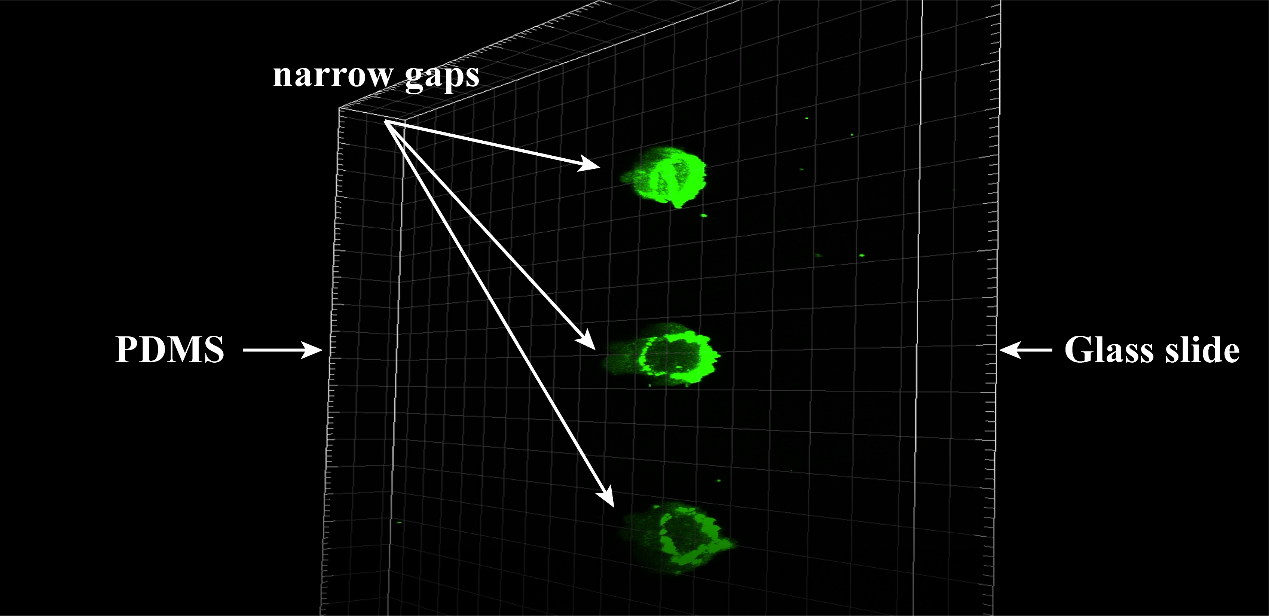


**Fig. S6.** The reconstructed 3D models from the confocal images of cancer cells after applying the given pressure 50 mbar for 120 min (green: FITC-vinculin).


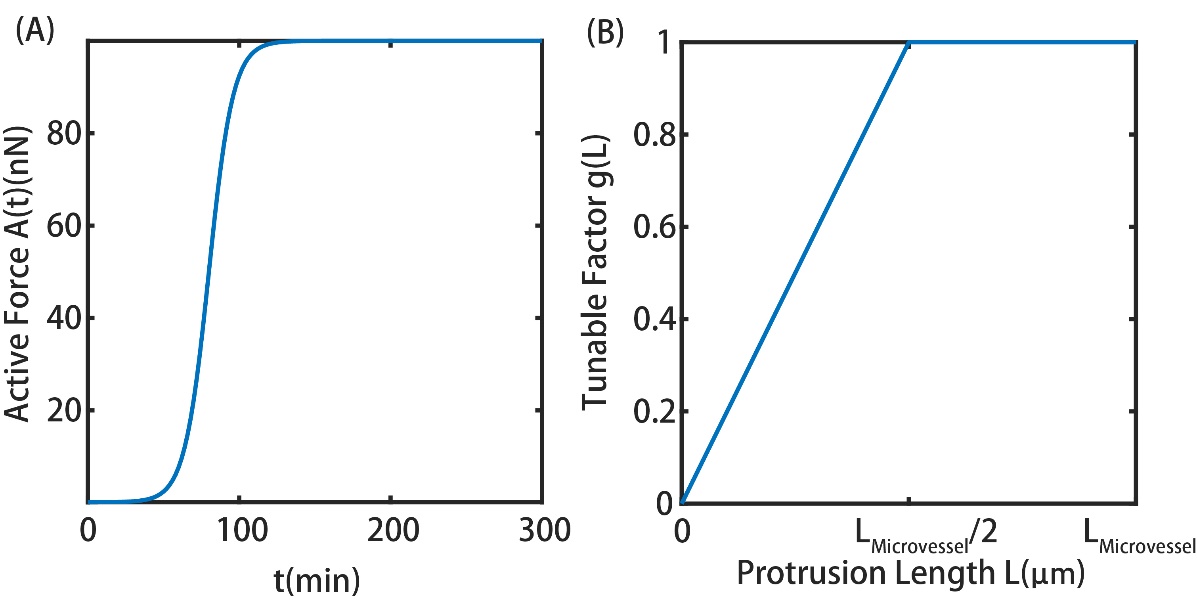


**Fig. S7.** (A) Active force *A*(*t*) as a function of time. (B) Tunable factor *g*(*L*) as a function of the protrusion length *L*.


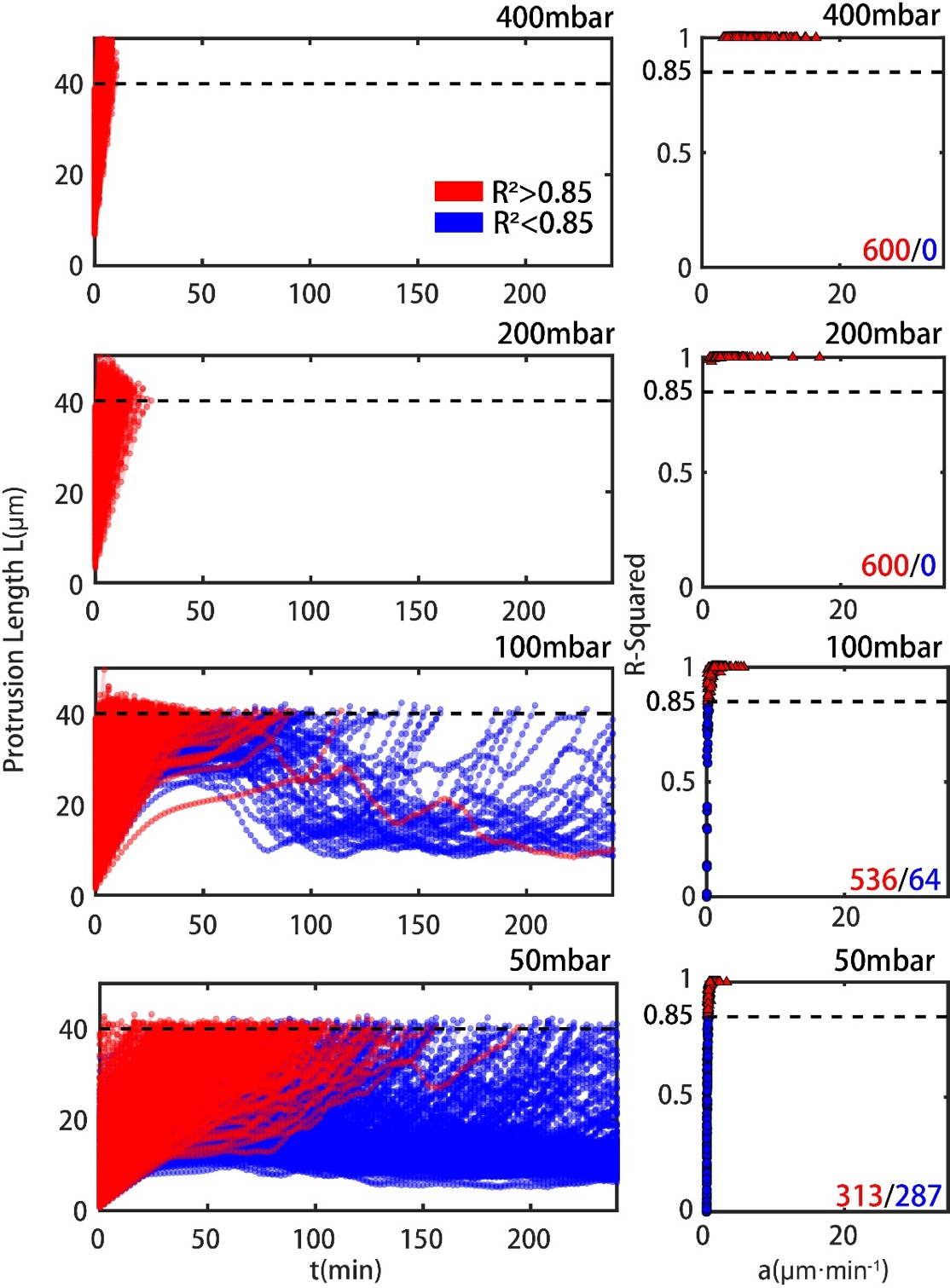


**Fig. S8.** Left: Simulation of dynamic behaviors under four applied pressure differences (400 mbar, 200 mbar, 100 mbar and 50 mbar) using the microfluidic chips with the size 7.5 μm×6 μm×40 μm of microvessels. Right: Scatter plot of R-squared values vs. fitting velocities for cells under four applied pressure differences (400 mbar, 200 mbar, 100 mbar and 50 mbar).


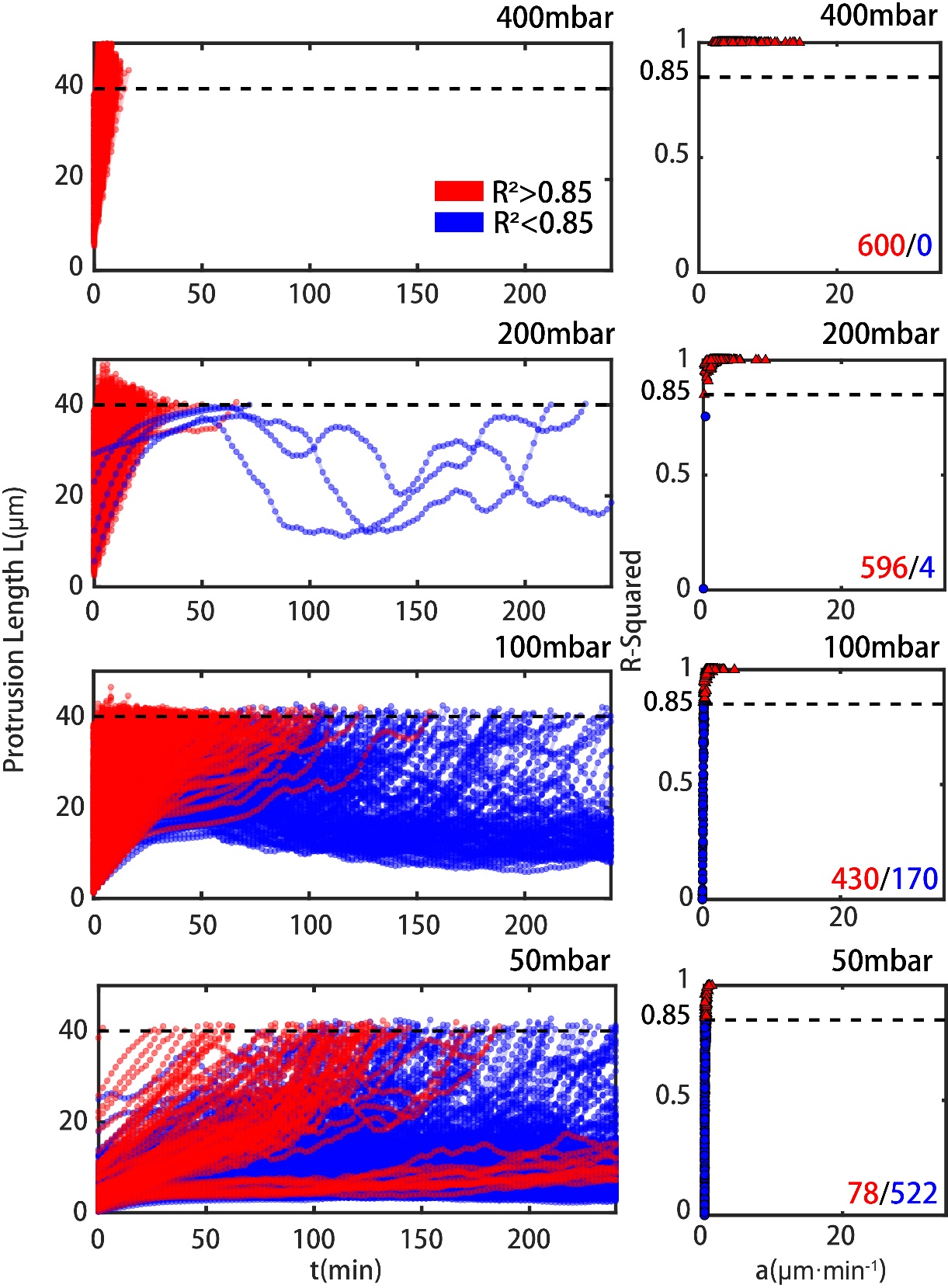


**Fig. S9.** Left: Simulation of dynamic behaviors under four applied pressure differences (400 mbar, 200 mbar, 100 mbar and 50 mbar) using the microfluidic chips with the size 6 μm×5 μm×40 μm of microvessels. Right: Scatter plot of R-squared values vs. fitting velocities for cells under four applied pressure differences (400 mbar, 200 mbar, 100 mbar and 50 mbar).


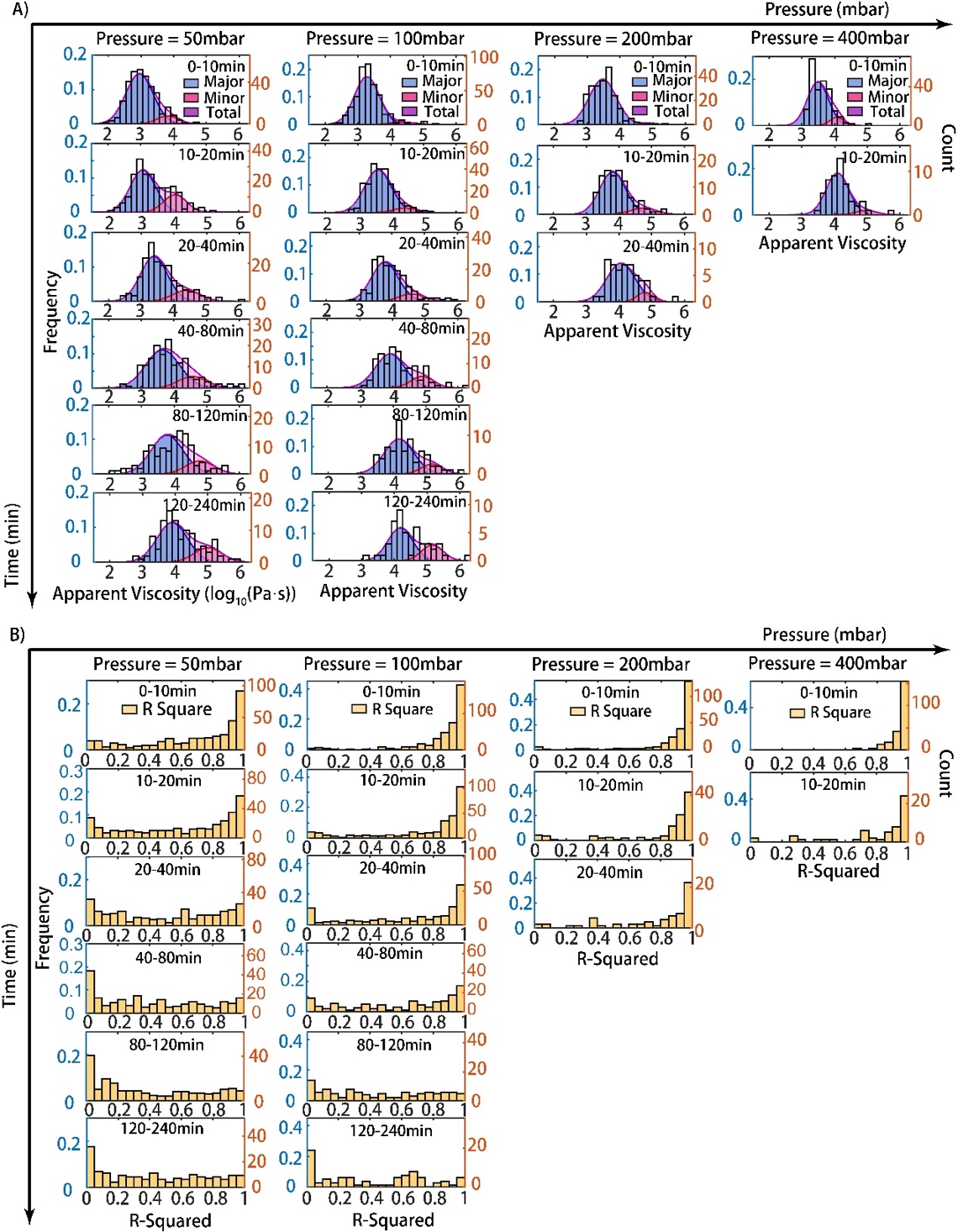


**Fig. S10.** Distribution analysis of cell behavior illustrated transitions for experiments. (A) Probability density distribution of cell apparent viscosities for 50, 100, 200 and 400 mbar at various periods (0-10, 10-20, 20-40, 40-80, 80-120, 120-240 mins). Major peaks in blue, minor peaks in pink and total peaks in purple. (B) Probability density distribution of the R-squared value of linear fitting for 50, 100, 200 and 400 mbar at various times. The left y-axes show frequencies, and the right y-axes give counts for both (A) and (B).


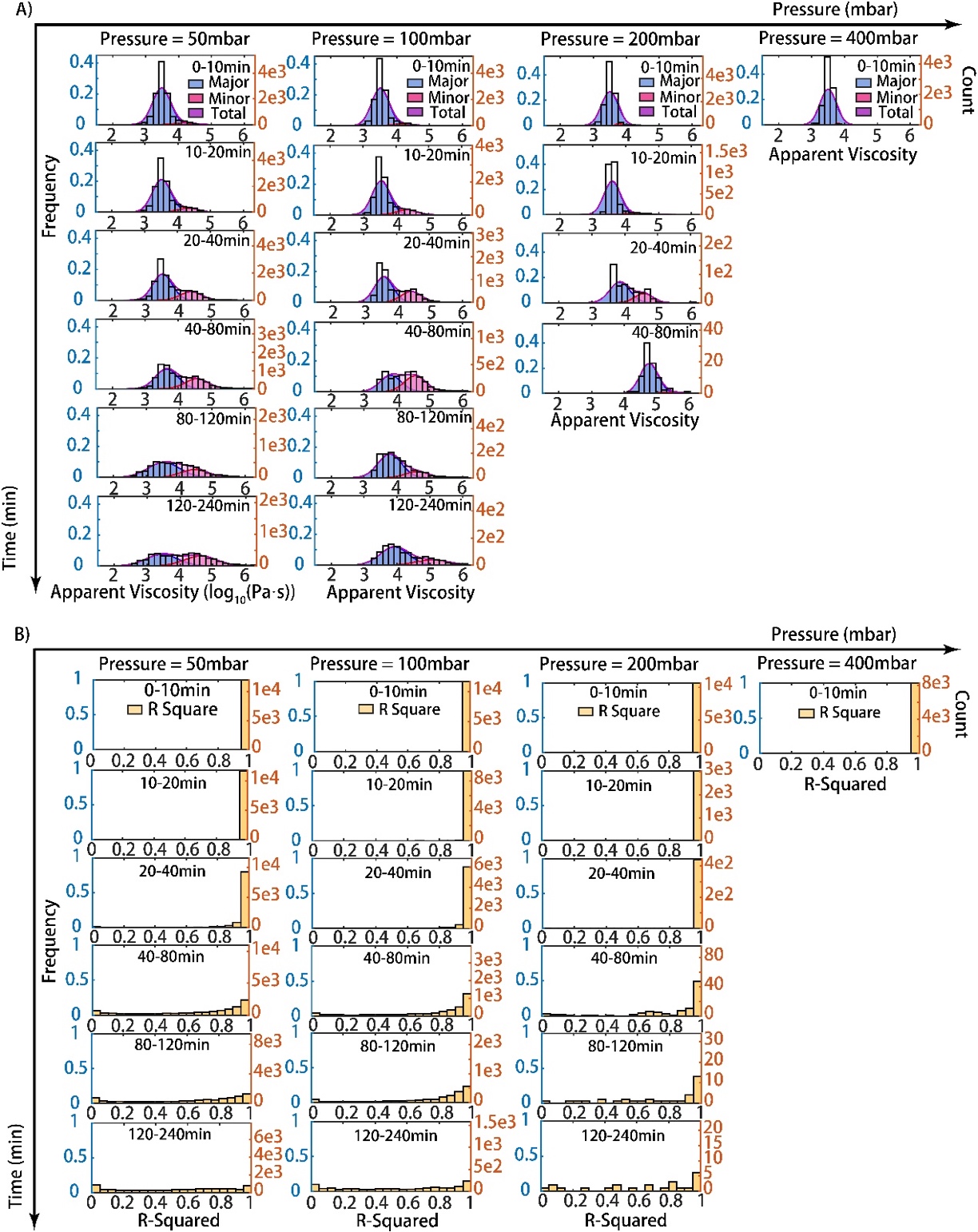


**Fig. S11.** Distribution analysis of cell behavior illustrated transitions for simulations. (A) Probability density distribution of cell apparent viscosities for 50, 100, 200 and 400 mbar at various periods (0-10, 10-20, 20-40, 40-80, 80-120, 120-240 mins). Major peaks in blue, minor peaks in pink and total peaks in purple. (B) Probability density distribution of the R-squared values of linear fitting for 50, 100, 200 and 400 mbar at various times. The left y-axes show frequencies, and the right y-axes give counts for both (A) and (B).


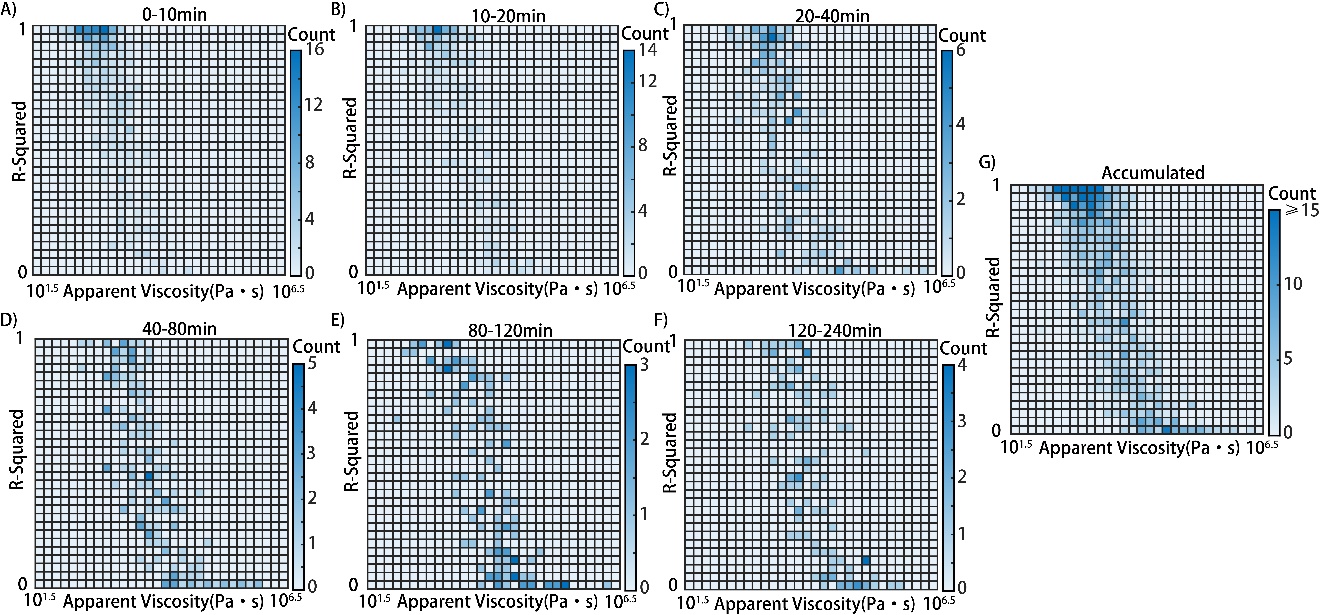


**Fig. S12.** (A-F) Heatmap depicting the number of cells with corresponding R-squared and apparent viscosities under a pressure difference of 50 mbar at all various periods in the experiments. (G) Heatmap depicting the accumulation of the numbers of cells with corresponding R-squared values and apparent viscosities under a pressure difference of 50 mbar in experiments.


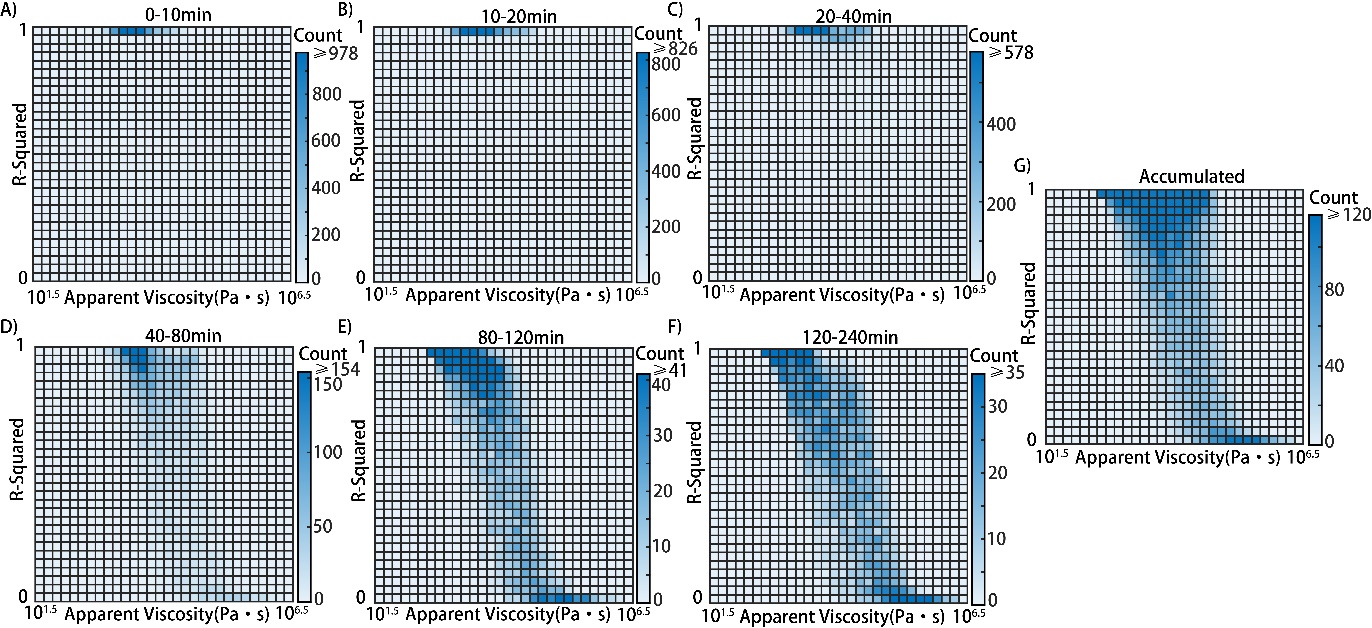


**Fig. S13.** (A-F) Heatmap depicting the number of cells with corresponding R-squared values and apparent viscosities under a pressure difference of 50 mbar at all various periods in simulations. (G) Heatmap depicting the accumulation of the numbers of cells with corresponding R-squared values and apparent viscosities under a pressure difference of 50 mbar in simulations.


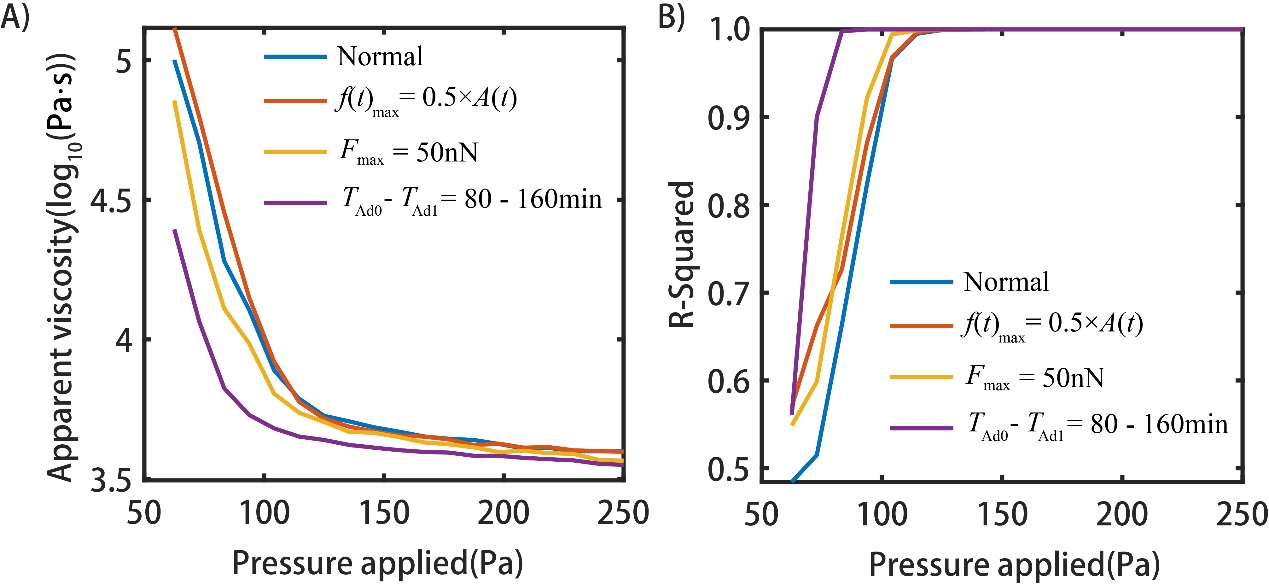


**Fig. S14.** Simulation results of apparent viscosities (A) and R-squared values (B) of cells under different pressure differences by varied parameters in the simulation (microvessel: 6 μm × 5 μm × 40 μm), which included four conditions: 1. Normal condition; 2. Set *f*(*t*)_max_=0.5×*A*(*t*), which means the maximum random force is half of the adhere force; 3. Set the Fmax to 50nN, which means the adherence force is decreased; 4. Set the *T*_ad0_ to 80 min and *T*_ad1_ to 160 min, which means the cell adherence process is inhibited.

**
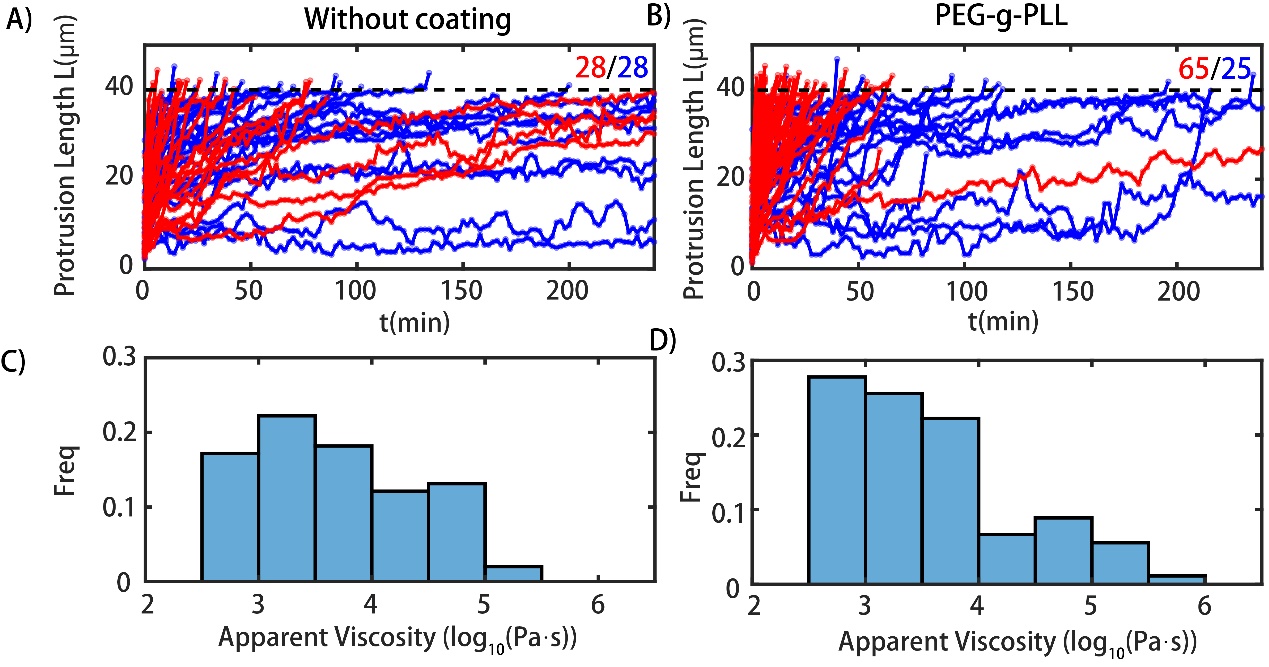
**

**Fig. S15.** Dynamic behaviors of MCF-7 cancer cells traversing microvessels 6 μm × 5 μm × 40 μm over time under pressure differences $\text{Δ}\text{P}$ of 100 mbar (A and C. without PLL-g-PEG coating; B and D. with PLL-g-PEG coating). Coating PLL-g-PEG to decrease the adherence effect of cancer cells shows that the traversing behavior seems to be more linear and that the apparent viscosities of cells are smaller than those in the uncoated situation. PLL-g-PEG Coating procedure as follow [16]**:** The synthetic copolymer, poly(L-lysine)-graft-poly(ethylene glycol) (PLL-g-PEG) was obtained as powders from Nanocs. The materials were stored at −20°C until use. The powder was then dissolved in 10 mM HEPES from Adamas at pH 7.4 to make stock solutions with a concentration of 1.0 mg/ml. Coating solution with a concentration of 0.1 mg/ml was created by mixing the stock solutions with 10 mM HEPES at pH 7.4. After degasing, 100μL of the coating solutions were pumped into microfluidic chips for 30 minutes, then were removed and the chips were washed with PBS.

**Table S1. Parameters used in simulations.**

| Name | Value | Source |
| --- | --- | --- |
| *μ* | 2675Pa·s | Determined by experiments. |
| *σ* | *μ*/3 | Obtained from previous studies [13]. |
| *F*_max_ | 100nN | Obtained from previous studies [13-14] |
| *F*_min_ | 0.1nN | Obtained from previous studies [13-14] |
| *T*_Ad0_ | 40min | Obtained from previous studies [15]. |
| *T*_Ad1_ | 120min | Obtained from previous studies [15]. |
| *k*_1_ | 10 | Derivated from (S6) and (S7) using *T*_Ad0_ and *T*_Ad1_. |
| *k*_2_ | 8 | Derivated from (S6) and (S7) using *T*_Ad0_ and *T*_Ad1_. |
| *n* | 3 | Speculation. |
| *R_f_ / S* | 30Pa (for 7.5×6μm) and 50Pa (for 6×5μm) | Determined by experiments. |
| *r* | 3.3μm (for 7.5×6μm) and 2.7μm (for 6×5μm) | Determined by experiments. |
| *α* | 40s/μm | Speculation. |

**Movie S1 (separate file). Movie of one typical cell under applied pressure difference $\text{Δ}\text{P}$, 200 mbar.**

**Movie S2(separate file) Movie of one typical cell under applied pressure difference $\text{Δ}\text{P}$, 100 mbar.**

**SI References**

[1] Chen, M., Lamar, J., Li, R., Hynes, R. & Kamm, R. Elucidation of the Roles of Tumor Integrin β1 in the Extravasation Stage of the Metastasis Cascade. *Cancer Research* 76, 2513-2524 (2016).

[2] Luo, C., Ni, X., Liu, L., Nomura, S. & Chen, Y. Degassing-assisted patterning of cell culture surfaces. *Biotechnology and Bioengineering* 105, 854-859 (2010).

[3] Curtis, A. Cell adhesion. *Progress in Biophysics and Molecular Biology* 27, 315-384 (1973).

[4] Kwon, K.W., et al. A microfluidic flow sensor for measuring cell adhesion. In *IEEE SENSORS 2006* 105-108 (IEEE, 2006).

[5] Komen, J. et al. Viability analysis and apoptosis induction of breast cancer cells in a microfluidic device: effect of cytostatic drugs. *Biomedical Microdevices* 10, 727-737 (2008).

[6] Kanidi, M. et al. Regulating MDA-MB-231 breast cancer cell adhesion on laser-patterned surfaces with micro- and nanotopography. *Biointerphases* 17, 021002 (2022).

[7] Mah, E., Lefebvre, A., McGahey, G., Yee, A. & Digman, M. Collagen density modulates triple-negative breast cancer cell metabolism through adhesion-mediated contractility. *Scientific Reports* 8, (2018).

[8] Razak, N. et al. Cytotoxicity of eupatorin in MCF-7 and MDA-MB-231 human breast cancer cells via cell cycle arrest, anti-angiogenesis and induction of apoptosis. *Scientific Reports* 9, (2019).

[9] Boyadzhieva, S. et al. A Self-Adhesive Elastomeric Wound Scaffold for Sensitive Adhesion to Tissue. *Polymers* 11, 942 (2019).

[10] Zhang, W., Choi, D., Nguyen, Y., Chang, J. & Qin, L. Studying Cancer Stem Cell Dynamics on PDMS Surfaces for Microfluidics Device Design. *Scientific Reports* 3, (2013).

[11] Evans, E. & Yeung, A. Apparent viscosity and cortical tension of blood granulocytes determined by micropipette aspiration. *Biophysical Journal* 56, 151-160 (1989).

[12] Needham, D. & Hochmuth, R. Rapid Flow of Passive Neutrophils Into a 4 μm Pipet and Measurement of Cytoplasmic Viscosity. *Journal of Biomechanical Engineering* 112, 269-276 (1990).

[13] Sagvolden, G., Giaever, I., Pettersen, E. & Feder, J. Cell adhesion force microscopy. *Proceedings of the National Academy of Sciences* 96, 471-476 (1999).

[14] Sabass, B., Gardel, M., Waterman, C. & Schwarz, U. High Resolution Traction Force Microscopy Based on Experimental and Computational Advances. *Biophysical Journal* 94, 207-220 (2008).

[15] Schlie, S., Gruene, M., Dittmar, H. & Chichkov, B. Dynamics of Cell Attachment: Adhesion Time and Force. *Tissue Engineering Part C: Methods* 18, 688-696 (2012).

[16] Norbert O., Beatrix P., Szilvia B., Jeremy J. R., Bálint S. & Robert H. Dependence of cancer cell adhesion kinetics on integrin ligand surface density measured by a high-throughput label-free resonant waveguide grating biosensor. *Scientific Reports* 4: 4034 (2014)
